# Cofactor Specificity of Glucose-6-Phosphate Dehydrogenase Isozymes in Pseudomonas putida Reveals a General Principle Underlying Glycolytic Strategies in Bacteria

**DOI:** 10.1128/mSystems.00014-21

**Published:** 2021-03-16

**Authors:** Daniel Christoph Volke, Karel Olavarría, Pablo Iván Nikel

**Affiliations:** a The Novo Nordisk Foundation Center for Biosustainability, Technical University of Denmark, Kongens Lyngby, Denmark; b Departamento de Microbiologia, Instituto de Ciências Biomédicas, Universidade de São Paulo, São Paulo, Brazil; Leiden University

**Keywords:** central carbon metabolism, cofactor specificity, glucose-6-phosphate dehydrogenase, glycolysis, *Pseudomonas putida*, redox balance

## Abstract

Glucose-6-phosphate dehydrogenase (G6PDH) is widely distributed in nature and catalyzes the first committing step in the oxidative branch of the pentose phosphate (PP) pathway, feeding either the reductive PP or the Entner-Doudoroff pathway. Besides its role in central carbon metabolism, this dehydrogenase provides reduced cofactors, thereby affecting redox balance. Although G6PDH is typically considered to display specificity toward NADP^+^, some variants accept NAD^+^ similarly or even preferentially. Furthermore, the number of G6PDH isozymes encoded in bacterial genomes varies from none to more than four orthologues. On this background, we systematically analyzed the interplay of the three G6PDH isoforms of the soil bacterium Pseudomonas putida KT2440 from genomic, genetic, and biochemical perspectives. P. putida represents an ideal model to tackle this endeavor, as its genome harbors gene orthologues for most dehydrogenases in central carbon metabolism. We show that the three G6PDHs of strain KT2440 have different cofactor specificities and that the isoforms encoded by *zwfA* and *zwfB* carry most of the activity, acting as metabolic “gatekeepers” for carbon sources that enter at different nodes of the biochemical network. Moreover, we demonstrate how multiplication of G6PDH isoforms is a widespread strategy in bacteria, correlating with the presence of an incomplete Embden-Meyerhof-Parnas pathway. The abundance of G6PDH isoforms in these species goes hand in hand with low NADP^+^ affinity, at least in one isozyme. We propose that gene duplication and relaxation in cofactor specificity is an evolutionary strategy toward balancing the relative production of NADPH and NADH.

**IMPORTANCE** Protein families have likely arisen during evolution by gene duplication and divergence followed by neofunctionalization. While this phenomenon is well documented for catabolic activities (typical of environmental bacteria that colonize highly polluted niches), the coexistence of multiple isozymes in central carbon catabolism remains relatively unexplored. We have adopted the metabolically versatile soil bacterium Pseudomonas putida KT2440 as a model to interrogate the physiological and evolutionary significance of coexisting glucose-6-phosphate dehydrogenase (G6PDH) isozymes. Our results show that each of the three G6PDHs in this bacterium display distinct biochemical properties, especially at the level of cofactor preference, impacting bacterial physiology in a carbon source-dependent fashion. Furthermore, the presence of multiple G6PDHs differing in NAD^+^ or NADP^+^ specificity in bacterial species strongly correlates with their predominant metabolic lifestyle. Our findings support the notion that multiplication of genes encoding cofactor-dependent dehydrogenases is a general evolutionary strategy toward achieving redox balance according to the growth conditions.

## INTRODUCTION

Glycolysis, the set of reactions that converts glucose and other sugars into biomass building blocks, is a ubiquitous metabolic feature across the tree of life. Glycolysis is often equated to the Embden-Meyerhof-Parnas (EMP) pathway, as it was first described in eukaryotes and the model Gram-negative bacterium Escherichia coli. However, glycolytic strategies are rather diverse, and several biochemical architectures (ranging from linear to highly interconnected cyclic networks) are deployed to metabolize glucose in different microbial species (1). Flamholz et al. (2) pointed out that there is no “superior” glycolysis (in terms of overall efficiency) and that each metabolic architecture can potentially confer survival advantages under certain environmental conditions. For instance, the Entner-Doudoroff (ED) pathway (and modified versions thereof) is a glycolytic strategy often found besides, instead of, or in combination with the EMP pathway (3–5). The ED pathway produces one ATP less per glucose than the EMP pathway (6), but it requires considerably less enzymatic resources (i.e., it is considered to be more efficient in terms of protein allocation). The ED pathway seems to prevail in bacteria with an aerobic lifestyle, where a large ATP fraction is generated by aerobic respiration instead of substrate-level phosphorylation (2). Besides providing precursors for biomass buildup, glycolytic routes recycle essential energy and redox cofactors, i.e., ATP, NADH, and NADPH. While ATP is mostly used as an energy currency, NADH and NADPH are carriers of reducing equivalents. Even though the chemical structures of NADH and NADPH are very similar, the NADH/NAD^+^ and NADPH/NADP^+^ redox ratios are very different, mirroring the different functions of these cofactors in the cell (7). NADH is primarily reoxidized in the respiratory chain to generate ATP, while NADPH drives anabolic reactions (8) and is also used to reduce glutathione as a first line of defense to oxidative stress (9).

Strategies to balance consumption and production of NADH and NADPH under different environmental conditions are a crucial trait for every cell. Besides altered cofactor specificities in dehydrogenases of central carbon metabolism, allosteric regulation mechanisms and rapid adjustment of carbon fluxes within the metabolic network are typically observed across bacterial species (10, 11). An important reaction for cofactor balance is catalyzed by glucose-6-phosphate (G6P) dehydrogenase (G6PDH), the first step of the oxidative pentose phosphate (PP) pathway that connects with the ED pathway. In the E. coli-centric (and prevailing) view of metabolism, this reaction is thought to serve as the primary source of NADPH (11–13), and the EMP pathway is the predominant catabolic route for hexoses in E. coli. Building on this notion, when the cofactor specificity of G6PDH of a given organism has not been characterized in detail, it is often assumed that it will prefer NADP^+^. However, several lines of evidence indicate that this is not the case for bacteria beyond E. coli (14, 15). For example, when this linear glycolytic route is blocked, e.g., in a Δ*pgi* mutant, and all the glycolytic flux is forced through the reaction catalyzed by G6PDH, the strict cofactor preference of G6PDH causes an NADPH imbalance in E. coli, leading to severe effects on bacterial growth (16, 17). In that case, mechanisms that prevent NADPH accumulation—e.g., overexpressing *sthA*, which encodes a soluble transhydrogenase (16), or substituting the native G6PDH by an NAD^+^-preferring orthologue (17)—partially rescue the growth rate of the Δ*pgi* mutant in cultures using glucose as the sole carbon source. This state of affairs prompts the following questions: what is the cofactor specificity of G6PDH in bacteria relying on the ED pathway for glycolysis, where most of the glycolytic flux is channeled through this dehydrogenase, and how do these bacteria adjust NADPH levels? Furthermore, what is the role of G6PDH in the NAD(P)H balancing mechanisms in those organisms? Studying the properties of G6PDHs in a model organism where these questions can be addressed is thus critical to understand the underlying regulatory mechanisms.

**FIG 1:**
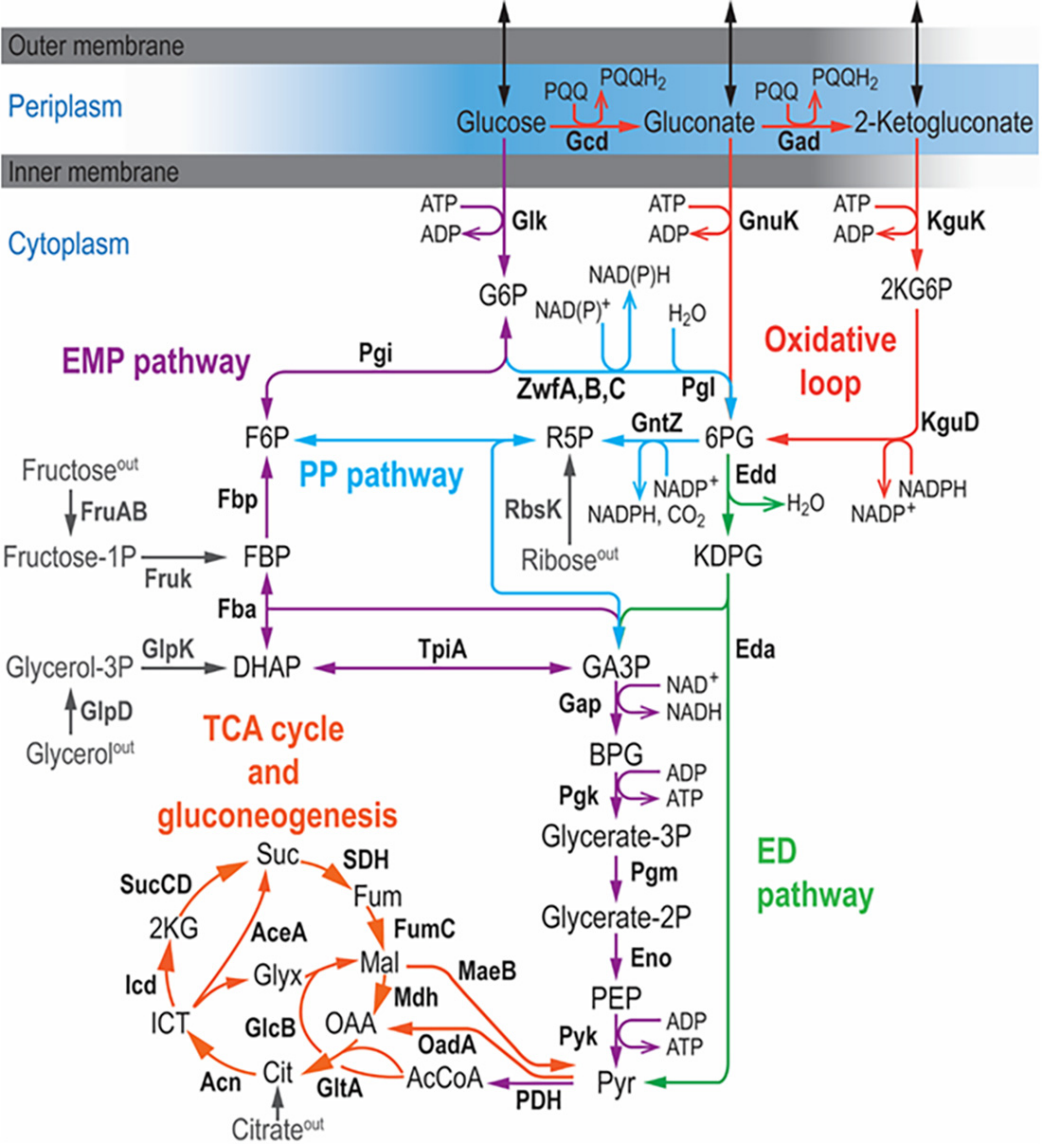
Organization of central carbon metabolism in P. putida KT2440. The metabolic network is sketched in six modules, depicted in different colors. Glucose is partly oxidized in the periplasm to yield gluconate and 2-ketogluconate, but both the sugar and its oxidized derivatives can be phosphorylated in the cytoplasm and metabolized through the Entner-Doudoroff (ED) pathway. Part of the carbon flux is cycled through the (incomplete) Embden-Meyerhof-Parnas (EMP) pathway operating in a gluconeogenic fashion, and a fraction of the glycolytic flux is channeled through the pentose phosphate (PP) pathway. The connecting point between the ED and EMP routes is the reaction catalyzed by glucose-6-phosphate dehydrogenases (G6PDH), encoded by three genes in strain KT2440: *zwfA*, *zwfB*, and *zwfC.* Furthermore, the upper domain glycolysis is connected to the tricarboxylic acid (TCA) cycle via the lower EMP pathway. For simplicity, some reactions are either lumped together or not shown in the diagram, and entry points of alternative carbon sources (i.e., fructose, ribose, glycerol, and citrate) are indicated in gray. Abbreviations for the key metabolites and intermediates in the network are as follows: 2KG6P, 2-keto-6-phosphogluconate; KDPG, 2-keto-3-deoxy-6-phosphogluconate; 6PG, 6-phosphogluconate; DHAP, dihydroxyacetone phosphate; FBP, fructose-1,6,-bisphosphate; GA3P, glyceraldehyde-3-phosphate; R5P, ribose-5-phosphate; BPG, 1,3-bisphosphoglycerate; PEP, phosphoenolpyruvate; Pyr, pyruvate; AcCoA, acetyl-coenzyme A; Cit, citrate; ICT, isocitrate; 2KG, 2-ketoglutarate; OAA, oxaloacetate; Suc, succinate; Fum, fumarate; Mal, malate; and Glyx, glyoxylate.

The use of the ED pathway is widespread in *Pseudomonas* and other bacteria (18, 19), and previous studies reported on the presence of several copies of G6PDH-encoding genes in this genus (20–23). Pseudomonas putida KT2440 and other members of this genus display a cyclic glycolysis (Fig. 1), employing the ED and PP pathways in combination with gluconeogenesis through the EMP route to oxidize hexoses (24). This structure, termed EDEMP cycle, was described in P. putida KT2440 as a biochemical mechanism that adjusts NADPH production levels by cycling the flux through upper glycolysis (24, 25). Besides this main set of reactions in the cytoplasm, P. putida harbors periplasmic enzymes for the direct oxidation of glucose to gluconate. Previous studies indicated that around 80% of glucose is taken up as gluconate or 2-ketogluconate, while the remaining 20% is phosphorylated to G6P (24, 26). G6PDH is a central enzymatic step for glycolysis in P. putida, and the three isoforms of this enzyme are encoded by *zwfA* (*PP_1022*), *zwfB* (*PP_4042*), and *zwfC* (*PP_5351*). G6PDH-A, encoded by *zwfA*, was shown to be highly produced under glycolytic conditions, with the transcription of the cognate gene (negatively) regulated by HexR (27). However, the functions (if any) of *zwfB* and *zwfC* and their corresponding products, G6PDH-B and G6PDH-C, remained elusive thus far.

Given such characteristics, we have adopted this bacterium as a model to explore the role of the different G6PDHs. We show that P. putida relies on the activity of isozymes with different cofactor specificities for the catabolism of several carbon sources. These enzyme variants were found to be especially important for growth on fructose and ribose, as nearly all the carbon flux goes through the G6PDH reaction. We also show that the presence of dual-cofactor and multiple G6PDH isozymes are a metabolic signature that strongly correlates with the use of the ED pathway as the main glycolytic strategy. As exposed by sequence analysis of G6PDHs across more than 1,000 bacterial species, this metabolic strategy appears to be widespread in the bacterial kingdom.

## RESULTS

### Growth patterns of single, double, and triple *zwf* mutants of P. putida KT2440 expose the physiological role of each isoform.

To shed light on the roles of the G6PDH isozymes, *zwfA*, *zwfB*, and *zwfC* were deleted in P. putida KT2440. The eight possible combinations of gene deletions comprising these three genes were constructed toward revealing the individual contribution of each variant to the overall physiology of the cells. These single and multiple *zwf* mutants were generated by markerless deletion based on homologous recombination (28, 29) and thoroughly checked for correctness by PCR amplification of the relevant loci and DNA sequencing (see Fig. S1 in the supplemental material).

10.1128/mSystems.00014-21.3 FIG S1 Confirmation of the genotypes of the *zwf* deletion strains. The genomic regions containing the *zwfA*, *zwfB*, or *zwfC* genes were amplified with the primer pairs *zwfA*-US-F/*zwfA*-DS-R, *zwfB*-US-F/*zwfB*-DS-R, and *zwfC*-US-F/*zwfC*-DS-R. A 1-kb gene ruler was used as ladder. WT, wild-type. Download FIG S1, PDF file, 0.3 MB. Copyright © 2021 Volke et al. 2021 Volke et al. https://creativecommons.org/licenses/by/4.0/ This content is distributed under the terms of the Creative Commons Attribution 4.0 International license.

**FIG 2:**
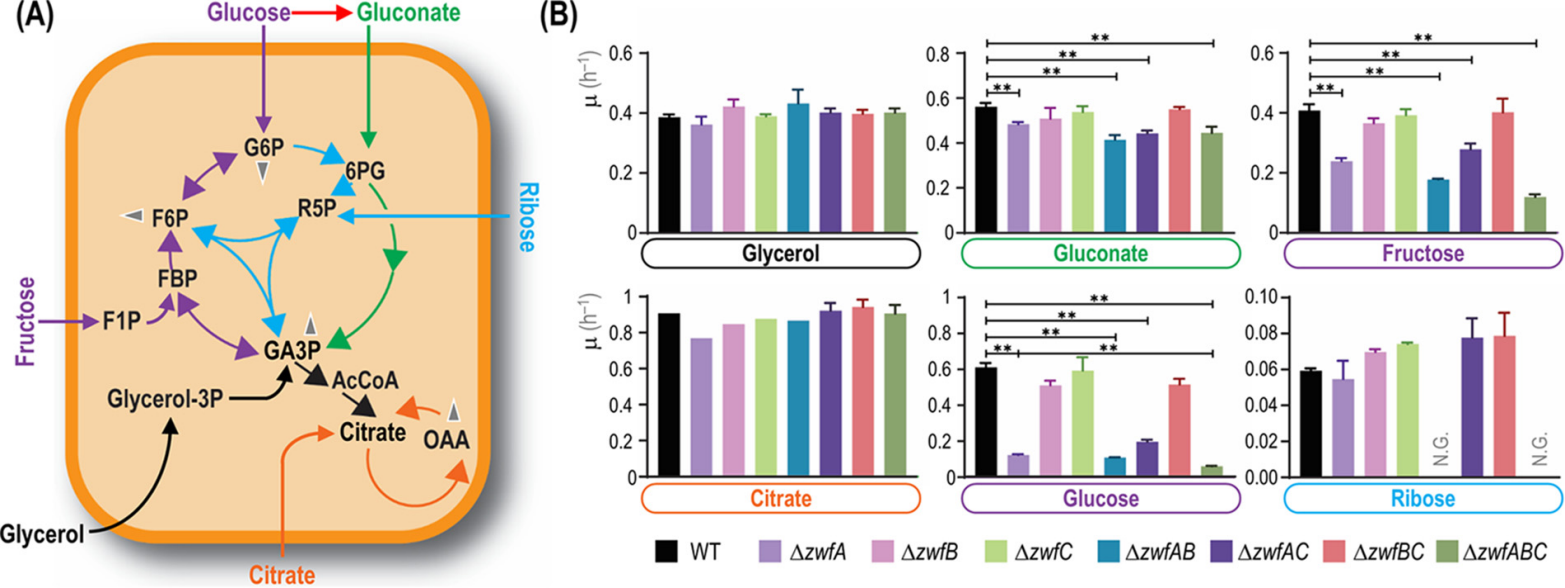
Analysis of growth patterns of different *zwf* mutants of P. putida KT2440. (A) Simplified scheme of the active metabolic blocks in the biochemical network of P. putida when cells were grown on the carbon sources indicated. Color codes and abbreviations are as indicated in the legend to Fig. 1. Glucose and fructose (C_6_) directly feed the (incomplete) EMP pathway, gluconate (C_6_) enters the ED route, and ribose (C_5_) is processed through the PP pathway. Citrate (C_6_) acts as an entirely gluconeogenic substrate, whereas glycerol (C_3_) is processed via both the upper gluconeogenic domain of the network and downward catabolism. Gray arrowheads indicate further conversion of key metabolic intermediates (e.g., used as precursors for biomass). (B) Batch cultures of wild-type (WT) strain KT2440 and all combinations of *zwf* deletion mutants were grown in M9 minimal medium supplemented with the substrates indicated such that they provided 120 mM total carbon. Carbon sources are identified with different colors according to the metabolic block they feed. Specific growth rates (μ) were determined during exponential growth, and bars represent the mean μ value ± standard deviation from three independent experiments per substrate. **, *P* < 0.01 assessed with the homoscedastic Student’s *t* test; N.G., no growth.

To test the coarse phenotype of the resulting strains, the mutants were first incubated under a number of cultivation conditions, and growth patterns were assessed. All mutant strains had the same specific growth rate (μ) when grown in complex lysogeny broth (LB) medium, indicating that mutations in *zwf* did not affect significantly cellular mechanisms involved in anabolism or bacterial division. Physiological characterization was also conducted in M9 minimal medium supplemented with different carbon sources (Fig. 2A), selected according to the catabolic modules involved in their processing. While glucose and gluconate are catabolized directly through activities within the EDEMP cycle (24), fructose is transported and phosphorylated by means of a dedicated sugar phosphotransferase (PTS) system (30), and ribose feeds the nonoxidative PP route (31). Citrate was chosen as an entirely gluconeogenic substrate (32), whereas glycerol enters at an intermediate point in the catabolic network of P. putida, triggering a mixed gluconeogenic and glycolytic processing regime (33). Glycerol goes into primary metabolism via its conversion to glycerol-3-phosphate (Fig. 2A) and then dihydroxyacetone phosphate (DHAP) and glyceraldehyde-3-phosphate (GA3P). From there, C_3_ units are metabolized either through the lower glycolysis (GA3P→pyruvate [Pyr]) or the gluconeogenic arm of the EMP pathway to generate intermediates with longer carbon backbones (GA3P→hexose phosphates) (33, 34). Citrate enters directly into the tricarboxylic acid (TCA) cycle (Fig. 2A), and besides direct oxidation, gluconeogenesis is used for biosynthesis of longer carbon chain metabolites (35).

All strains exhibited essentially the same growth patterns in glycerol and citrate cultures (Fig. 2B), with μ values similar to those reported previously (33). The apparent lack of any effect of *zwf* deletions on μ in glycerol cultures came as a surprise, since a major fraction of the carbon flux (73% to 104% of the glycerol uptake) is known to be routed through G6PDH (36) and also because the expression of the *zwfA-pgl-eda* operon is upregulated during growth of strain KT2440 on glycerol (27). These results indicate that P. putida is endowed with the ability to reroute the carbon flow during growth on glycerol, sustaining the supply of anabolic precursors produced in the PP pathway even when the G6PDH reaction is absent. On the other hand, D’Arrigo et al. (35) noted that in citrate-grown P. putida, fructose-6-phosphate (F6P) seems to enter the PP route directly rather than being converted into G6P and subsequently entering the oxidative phase of the PP pathway. Consistent with such an observation, the *zwf* mutants did not show differences in growth rates when grown on citrate (Fig. 2B).

In contrast to the results above, μ values differed across the strains tested when grown on glycolytic carbon sources. Mutants lacking *zwfA* displayed significant growth defects in cultures with gluconate (Fig. 2B), a substrate that is oxidized in the periplasm before entering the ED pathway downstream of G6PDH. However, no significant reduction in μ was observed for mutants lacking *zwfB*, *zwfC*, or both, indicating that the isoenzymes encoded by such genes do not display a significant role in gluconate metabolism. Gluconate assimilation highly induces the expression of genes in the HexR regulon (27), and as a consequence, *zwfA* expression is upregulated. Indeed, our results suggest that the activity of G6PDH-A alone can support all the required G6PDH flux under these cultivation conditions. Since no G6PDH activity is needed during the first steps of gluconate assimilation, the effect of deleting *zwfA* in gluconate cultures should be related to the insufficient G6PDH activity for flux cycling through the EDEMP pathway provided by G6PDH-B and G6PDH-C.

While the differences in gluconate-dependent growth of *zwfA* mutants were relatively minor, the growth of some knockout strains was severely impaired when cultivated on glucose or fructose. Besides a strong reduction in growth rates (e.g., μ of the Δ*zwfA* strain was 84% lower than μ for KT2440) (Fig. 2B), an extended lag phase was also observed in some cases (data not shown). Considering the high flux through the G6PDH reaction and the high level of expression of *zwfA* observed in P. putida when growing on glucose (27), it was not surprising to observe very low μ values in mutants lacking *zwfA*. According to metabolic fluxes estimations, most of glucose is first oxidized to gluconate (24, 26). Therefore, differences observed between growth patterns of *zwf* mutants on glucose (strong phenotype) or gluconate (mild phenotype) deserve special attention. A likely explanation stems from the steady state assumed for both flux balance analysis and ^13^C-based metabolic flux analysis, which differs from what actually happens during batchwise growth of P. putida without carbon limitation. Dynamic periplasmic conversion of glucose into gluconate and 2-ketogluconate affects flux distributions in the cytoplasm. As such, differences in μ values in gluconate and glucose cultures are connected to the complex dynamics of glucose assimilation (e.g., delayed gluconate uptake [37]). This occurrence could explain longer lag phases of glucose cultures as well as the larger impact of deleting *zwf* when the mutants are grown on glucose.

As indicated in Fig. 2A, fructose is phosphorylated to fructose-1-phosphate and subsequently to fructose-1,6-bisphosphate (FBP) (38). The single *zwfA* knockout displayed a significant decrease in μ (Fig. 2B), and even though the elimination of *zwfB* or *zwfC* did not result in major growth defects, the double Δ*zwfAB* mutant and the triple Δ*zwfABC* mutant displayed synergistic effects of individual deletions, with μ of the Δ*zwfABC* strain being 70% lower than μ of KT2440. As deduced from the observations in cultures using citrate or glycerol as the sole carbon sources, G6PDH activity can be fully substituted to feed the PP pathway. A possible explanation for the large growth difference of the same mutants on fructose compared to that on glycerol is metabolic imbalance—as P. putida utilizes FBP aldolase in the gluconeogenic direction (39, 40). Therefore, P. putida prioritizes the ED pathway for metabolization of fructose over direct formation of GA3P and DHAP (41). As the equilibrium of FBP aldolase is heavily displaced toward FBP (42), high FBP concentrations are needed to support glycolytic flux through the EMP pathway (43).

Finally, no growth of the double Δ*zwfAB* deletion mutant was observed on ribose (Fig. 2B), indicating that the catabolism of this pentose substrate also relies on the EDEMP cycle and suggesting that the G6PDH-C form has a near-zero activity. Because growth on ribose of wild-type P. putida was slow (μ ∼ 0.06 h^−1^), we confirmed the mutual essentiality of G6PDH-A and G6PDH-B by growing the Δ*zwfAB* strain on M9 minimal medium agar plates containing ribose for 180 h (see Fig. S2). As observed in the liquid medium experiments, no growth deficiency was observed for single knockouts, suggesting that the expression of either *zwfA* or *zwfB* ensures sufficient G6PDH activity to support the flux required for growth. This observation is in line with the expected catabolism of ribose, where two F6P molecules and one GA3P molecule are produced per three molecules of sugar. F6P cannot be metabolized further than G6P; therefore, F6P, G6P, and FBP may accumulate in this metabolic branch and will prevent further growth. In other *Pseudomonas* species, the buildup of F6P seems to be relieved by alginate accumulation (44), but such a mechanism is apparently not relevant in these strains. Since the analysis of single and combined *zwf* deletions indicated a differential role in the catabolism of glycolytic and gluconeogenic carbon sources, the next step was assessing the kinetic properties of each isoform.

10.1128/mSystems.00014-21.4 FIG S2 Growth phenotypes of *zwf* deletion strains on M9 minimal medium. The different *zwf* deletion strains were plated on agarized M9 minimal medium with the corresponding carbon source and incubated at 30°C for 24 h. Plates containing ribose were incubated for 180 h to compensate for the slow growth of some of the mutant strains under these conditions. Download FIG S2, PDF file, 0.3 MB. Copyright © 2021 Volke et al. 2021 Volke et al. https://creativecommons.org/licenses/by/4.0/ This content is distributed under the terms of the Creative Commons Attribution 4.0 International license.

### Kinetic properties of individual G6PDH isoforms of P. putida KT2440 indicate different physiological roles.

Although most G6PDH are described as NADP^+^-dependent enzymes, previous reports suggested that dual or even NAD^+^-preferring homologues exist in several bacterial species, including *Pseudomonas* and close relatives (20–22, 45–47). The kinetic properties of the G6PDH-B and G6PDH-C isoforms present in strain KT2440 have not been studied so far, and whether G6P oxidation catalyzed by these variants would funnel electrons into the NADH or NADPH pools remains unclear. Because each redox cofactor has a distinct metabolic function, we reasoned that assessing the relative production of nucleotides by different G6PDHs *in vitro* would help understanding the presence of isoforms and their respective roles in P. putida. To this end, the genes encoding G6PDH-B and G6PDH-C were cloned and expressed in E. coli, and the enzymes were purified to homogeneity by Ni^2+^-based affinity chromatography. The kinetic properties of these purified G6PDH variants were then studied using either NAD^+^ or NADP^+^ as the redox cofactors. The key biochemical parameters for G6PDH-A, G6PDH-B, and G6PDH-C, including the turnover constant (*k*_cat_) and the Michaelis constant (*K_m_*) values for each variant were calculated based on Michaelis-Menten plots (see Fig. S3) and are reported in Table 1.

**TABLE 1:** Kinetic parameters of the three G6PDH isoforms in P. putida KT2440^*a*^

Isozyme	NAD^+^					NADP^+^					Cofactor specificity constant (φ)
	*K*_cat_ (s^–1^)	*K_m_* (μM)	*K_i_* (μM)	*K_m_* G6P (μM)	*K_ic_* (μM)	*K*_cat_ (s^–1^)	*K_m_* (μM)	*K_i_* (μM)	*K_m_* G6P (μM)	*K_ic_* (μ)	
G6PDH-A^*b*^	277 ± 2	127 ± 8	1148 ± 67	1137 ± 37	480 ± 7	102 ± 1	14 ± 2	111 ± 12	946 ± 49	18 ± 3	3.34 ± 0.52
G6PDH-B	120 ± 1	151 ± 7	301 ± 48	291 ± 27	337 ± 43^*c*^^*d*^	113 ± 1	165 ± 11	790 ± 110	190 ± 15	38 ± 6^*d*^	0.86 ± 0.07
G6PDH-C	0.77 ± 0.04	(9.5 ± 1.1)×10^3^	(5.8 ± 1.2)×10^3^	2,030 ± 220	(23 ± 4)×10^6^^*d*^	0.54 ± 0.02	3.2 ± 0.7	9.2 ± 1.9	944 ± 84	775 ± 255^*d*^	2,082 ± 532

a Abbreviations used in the table are as follows: *k*_cat_, turnover constant; *K_m_*, Michaelis constant; *K_i_*, dissociation constant for the interaction NAD(P)^+^ + free enzyme ↔ enzyme-NAD(P)^+^ complex; and *K_ic_*, NAD(P)H competitive inhibition constant. The cofactor specificity constant (φ) for each variants was calculated as [*k*_cat_NADP^+^/*K_m_*NADP^+^]/[*k*_cat_NAD^+^/*K_m_*NAD^+^].

b Experimental data taken from Olavarría et al. (22).

c NAD^+^ also had an inhibitory effect on the activity of G6PDH-B, with an inhibition constant *K_is_* of 1,390 ± 220 μM.

d Values estimated using Haldane’s relationships.

10.1128/mSystems.00014-21.5 FIG S3 Michaelis-Menten plots for G6PDH-B and G6PDH-C of P. putida KT2440. Kinetic parameters (initial conversion rate, *V*_0_) of G6PDH-B were measured in the presence of NAD^+^ (A) and NADP^+^ (B) as cofactors. Likewise, the kinetics of G6PDH-C were assessed with NAD^+^ (C) and NADP^+^ (D) as cofactors. Different cofactor and glucose-6-phosphate (G6P) concentrations (concn.) were used as indicated in the individual plots. Three independent determinations (and their technical replicates) are shown either as individual datapoints (for G6PDH-B) or as averages (for G6PDH-C). Download FIG S3, PDF file, 0.3 MB. Copyright © 2021 Volke et al. 2021 Volke et al. https://creativecommons.org/licenses/by/4.0/ This content is distributed under the terms of the Creative Commons Attribution 4.0 International license.

The rapid-equilibrium random-ordered mechanism was the best model to explain the results observed in all cases, similar to previous findings reported for the G6PDH-A isoform of strain KT2440 (22). Importantly, the main differences between G6PDH-A and G6PDH-B were detected in the *K_m_* values for NADP^+^ and G6P, which indicate that the response of these enzymes to changes in the cytoplasmic concentrations of these metabolites could be different (Table 1). The cofactor specificity constants (φ, defined as [*k*_cat_NADP^+^/*K_m_*NADP^+^]/[*k*_cat_NAD^+^/*K_m_*NAD^+^]) were clearly different with respect to the φ of well-characterized NADP^+^-preferring homologues. G6PDH-C had a very different set of kinetic parameters than the other two isozymes: this isozyme exhibited the highest *K_m_* (>10^3^ μM) for NAD^+^ and the lowest *K_m_* for NADP^+^ (<10 μM) among the three G6PDHs. Therefore, G6PDH-C shows a very high specificity for NADP^+^ (as a comparison, G6PDH from E. coli, widely regarded as an NADP^+^-preferring dehydrogenase [48], is reported to have a φ of 410, while φ of G6PDH-C is 5-fold higher than that value). However, it exhibited a very slow turnover (*k*_cat_ < 1 s^−1^) in the presence of both NAD^+^ and NADP^+^. Such a low turnover constant is consistent with the negligible effect of deleting *zwfC* on μ under all the cultivation conditions tested (Fig. 2B).

**FIG 3:**
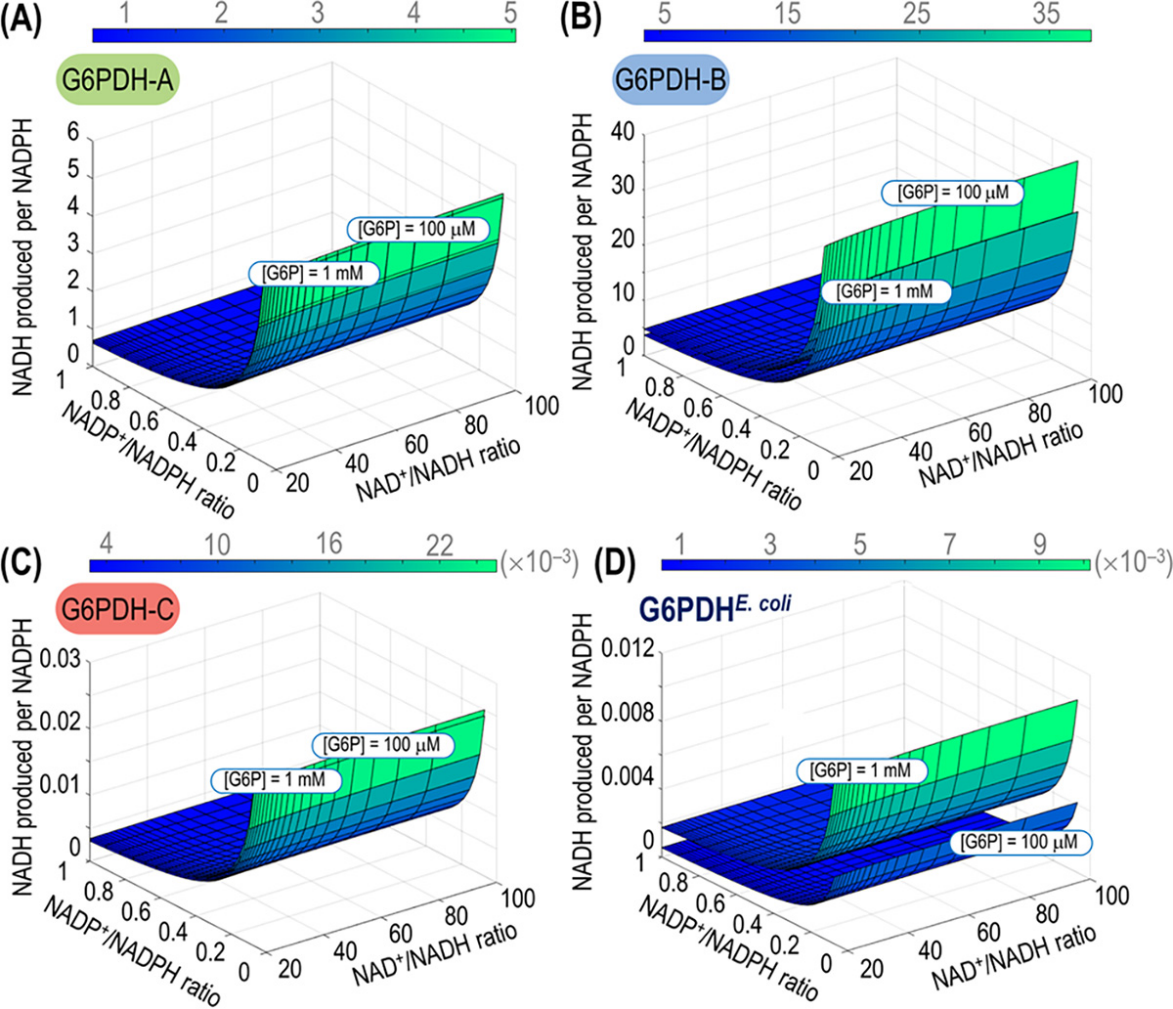
Relative NADH-to-NADPH output of the G6PDH isozymes of P. putida KT2440. The relative production of the two reduced cofactors was simulated across physiologically relevant NAD^+^/NADH and NADP^+^/NADPH ratios and two glucose-6-phosphate (G6P) concentrations for G6PDH-A (A), G6PDH-B (B), and G6PDH-C (C). (D) The relative NADH-to-NADPH production was also simulated for the single G6PDH enzyme of E. coli, included here for the sake of comparison.

Considering the results above and, especially, the differences in φ values among the dehydrogenase variants, we addressed the question of cofactor specificity under *in vivo*-like conditions. Although φ provides a solid indication of cofactor preference under saturating conditions, physiologically relevant circumstances are dynamic and often different from the setup adopted for *in vitro* kinetic assays. One of the most important differences *in vivo* is the simultaneous presence of NAD^+^, NADP^+^, NADH, and NADPH, besides various concentrations of G6P and other potential effectors not added to the *in vitro* assays. Thus, we adopted a simplified kinetic model to evaluate the relative production of NADPH and NADH by the G6PDH isozymes under study under physiologically relevant conditions, i.e., applying variable G6P concentrations and NAD^+^/NADH and NADP^+^/NADPH redox ratios consistent with the thermodynamic constrains enabling the operation of metabolism (Fig. 3). Significant differences between the G6PDH isoforms were observed when the relative NADH-to-NADPH output was simulated as a function of the redox ratios at two G6P concentrations spanning 1 order of magnitude (i.e., 100 μM and 1 mM, compatible with values reported for glucose-grown strain KT2440 [24, 25]). While the activity of G6PDH-A yielded essentially the same amounts of NADH and NADPH regardless of the G6P concentration (NADH produced per NADPH close to 1) (Fig. 3A), G6PDH-B generated mostly NADH, and the relative production of NADH over that of NADPH showed a higher dependence on the G6P concentration (Fig. 3B). The high sensitivity of G6PDH-B with respect to substrate availability comes from a *K_m_* for G6P 5-fold lower than that of G6PDH-A, either with NAD^+^ or NADP^+^ as the cofactor (Table 1). G6PDH-C, in turn, has the lowest NADH-to-NADPH output across the experimental conditions simulated (Fig. 3C), in line with its high φ. The notion that NADP^+^ is the preferred cofactor of the archetypal G6PDH of E. coli became evident when its NADH-to-NADPH output profile was compared to G6PDH-A and G6PDH-B (Fig. 3D). Moreover, the relative NADH over NADPH production of G6PDH of E. coli had a more pronounced dependence on the G6P concentration. These biochemical features indicate different roles in the cellular redox balance for the different G6PDH isozymes. Yet, the regulatory pattern of the cognate genes (and, in particular, that of *zwfC*) remains unclear, and we set out to investigate this aspect as disclosed in the next section.

### The *zwfC* gene from P. putida KT2440 is poorly transcribed across different growth conditions.

**FIG 4:**
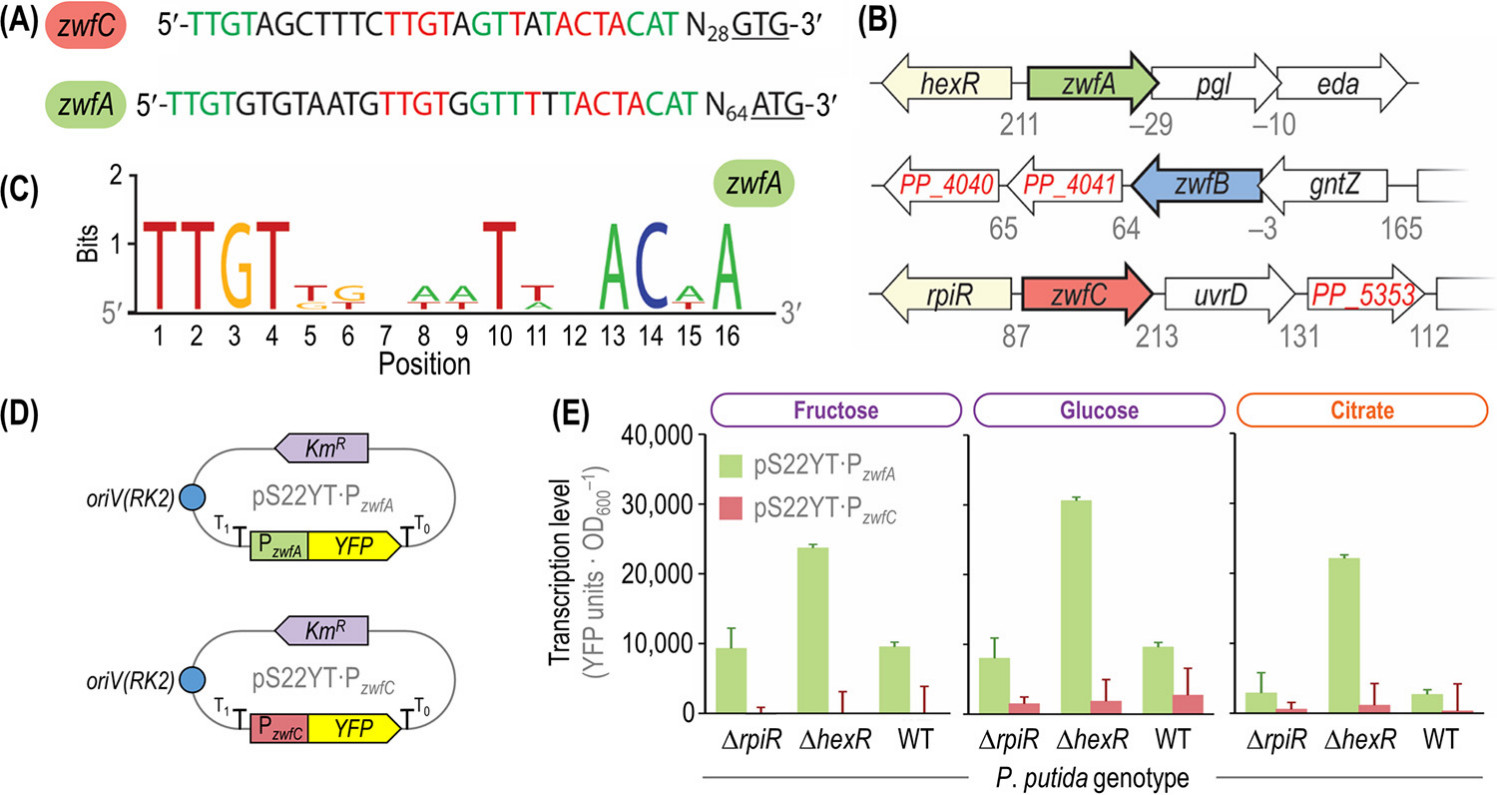
Genetic and genomic organization of *zwf* orthologues in P. putida KT2440 and analysis of transcriptional levels. (A) Upstream region of the *zwfA* and *zwfC* genes. Both regions contain a duplicated HexR-binding site (marked in red) and share sequences with high similarity (indicated in green) as annotated by Belda et al. (95). The start codons of *zwfA* and *zwfC* (ATG and GTG, respectively) are underlined, and N represents any nucleotide. (B) Genomic organization of the three *zwf* orthologues of strain KT2440. The *zwfA* gene forms an operon together with *pgl* and *eda* (encoding 6-phosphogluconolactonase and 2-keto-3-deoxy-6-phosphogluconate aldolase, respectively), and its expression is controlled by the HexR regulator. The *zwfB* gene is cotranscribed with *gntZ* (encodes 6-phosphogluconate dehydrogenase), while *zwfC* is divergently transcribed with respect to *rpiR* (PP_5050, a transcriptional regulator). Numbers below the gene clusters indicate intergenic distances in base pairs, with negative values representing an overlap between the corresponding open reading frames. Elements in this diagram are not drawn to scale. (C) Consensus sequence of HexR-binding sites for *zwfA*, *gapA* (encodes glyceraldehyde-3-phosphate dehydrogenase), and *edd* (encodes 6-phosphogluconate dehydratase). (D) Reporter plasmids constructed to assess *zwfA* and *zwfB* expression levels. The promoter region of the corresponding genes was cloned in front of the gene encoding the yellow fluorescent protein (YFP), and the resulting modules are transcriptionally insulated by means of the T_0_ and T_1_ terminators. These low-copy-number [*oriV*(RK2)] reporter plasmids are endowed with a kanamycin resistance (Km^r^) cassette. (E) Transcriptional analysis of the P*_zwfA_* and P*_zwfC_* promoters in different genetic backgrounds. Wild-type (WT) P. putida KT2440 and its Δ*hexR* or Δ*rpiR* mutant derivatives were transformed with the reporter plasmids described for panel D, carrying the promoter regions of *zwfA* and *zwfC*. Fluorescence values were normalized against those in the WT strain carrying vector pSEVA227Y (promoterless YFP). Carbon sources are identified with different colors according to the metabolic block they feed (see Fig. 2). Bars represent mean values ± standard deviations from three independent experiments per substrate. OD_600_, optical density measured at 600 nm.

According to the experiments described in the preceding sections, the deletion of *zwfC* has no significant effect on the growth patterns in any of the carbon sources tested (Fig. 2B). Therefore, we wondered whether *zwfC* encodes a functional G6PDH enzyme, considering that a purified G6PDH-C showed the highest specificity for NADP^+^ *in vitro* (Table 1). According to the genome annotation, *zwfC* encodes a nontruncated G6PDH-like protein. Additionally, an alignment of G6PDH-C with the G6PDH enzyme from Leuconostoc mesenteroides revealed that both the cofactor binding motif (G_12_-[G/A]-X-GDL-[A/V]-[K/L] at the N terminus, with X representing any amino acid) and other residues key for the interaction with substrates (i.e., R[176]IDHYLGKE, E[146]KP, Y415, and H240) are conserved. However, its expression and/or activity could be further modulated by transcriptional, translational, or posttranslational mechanisms that could render the gene or the polypeptide silent. To explore this possibility at the transcriptional level of regulation, we first searched for ribosomal binding sites (RBS) upstream of the open reading frame of *zwfC* by using the RBS calculator tool (49). Several in-frame START codons, with a predicted medium-to-strong translation efficiency, were found around the annotated start of the open reading frame according to this analysis. Second, we looked for potential promoter regions upstream of the G6PDH-C-encoding sequence. Interestingly, the 5′ untranslated region of *zwfC* has a duplicated HexR binding motif, highly similar to the recognition motif found upstream of *zwfA* (Fig. 4A). This motif follows the canonical consensus HexR binding site (Fig. 4B), originally identified by Daddaoua et al. (50) not only for *zwfA* but also for *gapA* and *edd*. Furthermore, upstream of *zwfC* there is a divergent gene, *PP_5350* (867 bp), predicted to encode a transcriptional regulator of the RpiR family (Fig. 4C). This RpiR regulator shares a high similarity with HexR (45% identity; *hexR* is 864 bp long), and the binding moieties for sugars (residues R53 and R56) as well as for DNA (residues S139 and S183) are conserved (51). Interestingly, such genomic organization is not exclusive of P. putida, and we have identified orthologues of *rpiR* and *zwfC* adjacent to each other in many annotated genomes of *Pseudomonas* and closely related species (representative examples are shown in Fig. S4). Such architecture, where the gene encoding a putative transcriptional regulator is located close to the regulated gene(s), is widespread in nature.

10.1128/mSystems.00014-21.6 FIG S4 Comparison of the genomic organization around *zwfC* in different species. The cladogram was constructed based on the nucleotide sequence of *gyrB* using maximum likelihood analysis in MEGA. The corresponding distance is annotated next to the branches of the tree. The comparison of the genomic regions adjacent to *zwfC* was composed by CLINKER. Download FIG S4, PDF file, 0.3 MB. Copyright © 2021 Volke et al. 2021 Volke et al. https://creativecommons.org/licenses/by/4.0/ This content is distributed under the terms of the Creative Commons Attribution 4.0 International license.

To investigate the potential role of RpiR on *zwfC* expression, we constructed a set of transcriptional reporter plasmids (Fig. 4D) derived from the low-copy-number pSEVA227Y vector (52). Plasmid pS22YT·P*_zwfC_* contains the 278-bp-long 5′ untranslated region (UTR) of *zwfC* (spanning the predicted promoter sequence) in front of the gene encoding the yellow fluorescent protein (YFP). The upstream region of *zwfA* was also cloned in the same backbone, yielding plasmid pS22YT·P*_zwfA_*, where the promoter region of *zwfA* drives the expression of *YFP*. In addition, we created individual Δ*rpiR* and Δ*hexR* mutant derivatives of P. putida KT2440 (see Table S1). These deletion mutants, together with the wild-type strain, were transformed with the reporter plasmids or the empty pSEVA227Y vector and cultivated to measure the signal of the reporter protein across growth conditions. No significant *YFP* expression from P*_zwfC_* was detected in the wild-type strain or in the *ΔhexR* or *ΔrpiR* mutants when grown on fructose (Fig. 4E). Detectable expression levels from the P*_zwfA_* promoter were observed in all genetic backgrounds, with a strong (2.5-fold) increase in YFP fluorescence in the *ΔhexR* mutant compared to that in P. putida KT2440 (Fig. 4E). A similar trend was also observed in glucose cultures. In this case, and even though expression from P*_zwfC_* was marginally higher in all the tested strains, the differences in YFP levels in the wild-type strain and the mutants were not significant (Fig. 4E). When the experiments were repeated in the presence of citrate, a gluconeogenic carbon source, almost no expression from P*_zwfC_* was observed in any of the genetic backgrounds tested. The normalized YFP signal triggered by P*_zwfA_* in citrate cultures, in contrast, was around half as strong as that observed in the wild-type strain and the *ΔrpiR* mutant on fructose and similarly high as in the *ΔhexR* mutant grown on fructose (Fig. 4E). In agreement with these observations, low *zwfC* mRNA levels were detected by deep sequencing of the transcriptome of strain KT2440 grown on several carbon sources (53), while the highest *zwfA* transcription levels were observed on glucose (see Fig. S5). Taken together, these results indicate that the levels of *zwfC* expression are extremely low under either glycolytic or gluconeogenic regimes and that the regulatory role of RpiR (if any) does not seem to be relevant under the experimental conditions tested. The next question was assessing the actual G6PDH activity in the different *zwf* mutants.

10.1128/mSystems.00014-21.1 TABLE S1 Bacterial strains and plasmids used in this study. Download Table S1, PDF file, 0.7 MB. Copyright © 2021 Volke et al. 2021 Volke et al. https://creativecommons.org/licenses/by/4.0/ This content is distributed under the terms of the Creative Commons Attribution 4.0 International license.

10.1128/mSystems.00014-21.7 FIG S5 Transcriptional levels of *zwfA, zwfB*, and *zwfC* in P. putida KT2440 growing on different carbon sources. Data taken from the RNA sequencing (RNA-Seq) analysis published by Kim et al. (53). Download FIG S5, PDF file, 0.2 MB. Copyright © 2021 Volke et al. 2021 Volke et al. https://creativecommons.org/licenses/by/4.0/ This content is distributed under the terms of the Creative Commons Attribution 4.0 International license.

### G6PDH-A provides the bulk of the glucose-6-phosphate dehydrogenase activity in glucose-grown P. putida KT2440.

**FIG 5:**
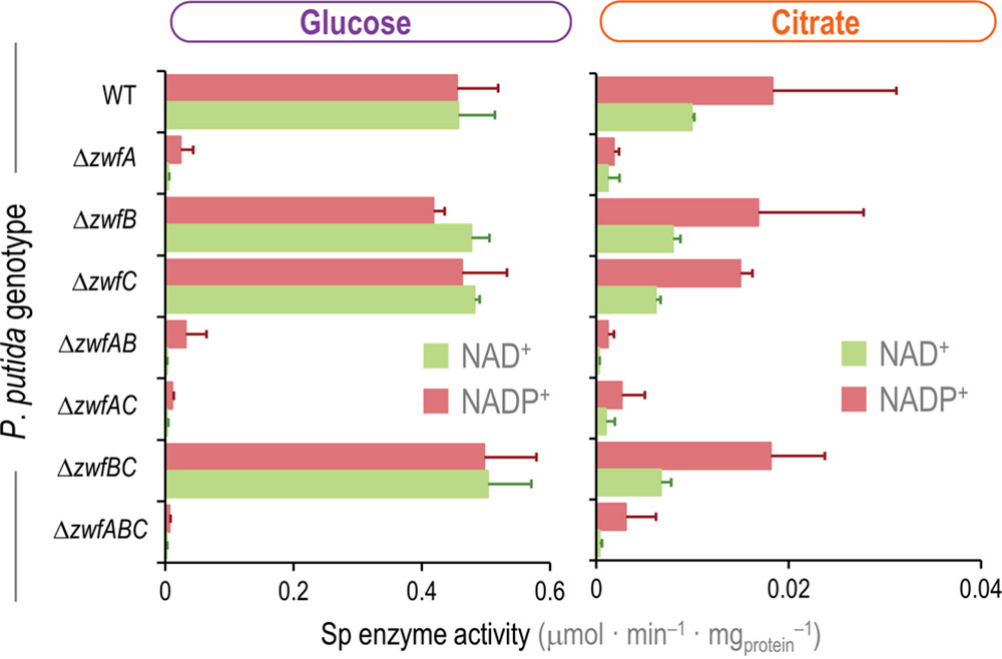
*In vitro* determination of glucose-6-phosphate dehydrogenase activity in P. putida KT2440 and the different *zwf* knockouts. The specific (Sp) G6PDH activity was determined *in vitro* in cell extracts obtained from glucose or citrate cultures, assayed in the presence of either NAD^+^ or NADP^+^ as indicated. Carbon sources are identified with colors according to the metabolic block they feed (see Fig. 2A). Data represent the mean values of the specific enzymatic activity ± standard deviations from triplicate measurements from at least three independent experiments.

The physiological data (Fig. 2), the *in vitro* biochemical characterization of purified enzymes (Table 1), and the transcriptional pattern (Fig. 4) strongly suggest that G6PDH-C does not carry a significant flux, which prompts the question of which isozyme(s) actually provides the G6PDH activity in the strain KT2440. To this end, the G6PDH activities of all constructed *zwf* mutant strains were assayed in cell extracts obtained from glucose and citrate cultures (Fig. 5). Moreover, we tested enzyme activities using either NAD^+^ or NADP^+^ as the cofactor added to the reaction mixture. As expected, the overall G6PDH activity was consistently higher (around 1 order of magnitude) in cells grown under a glycolytic regime than in those grown under gluconeogenic conditions. The specific activity was almost lost in mutants deficient in G6PDH-A when grown on glucose, indicating that this isozyme supplies by far the most activity under glycolytic conditions. The specific G6PDH activities in the wild-type strain were similar to previous reports (24, 25), and the activity observed in cell extracts of the Δ*zwfABC* triple mutant was very low, indicating a negligible level of promiscuous NADP^+^-dependent dehydrogenases that could interfere in this assay (Fig. 5). No activity above the level of the Δ*zwfABC* mutant could be detected in extracts of the Δ*zwfAB* double mutant, indicating, again, that G6PDH-C does not supply a significant activity under the conditions tested. Interestingly, in glucose-grown cells, the G6PDH activity was essentially the same regardless of the cofactor used in the reactions, similar to the simulations shown in Fig. 3A, pointing to the fact that G6PDH-A is the predominant isozyme under such conditions. The bulk G6PDH activity in citrate-grown P. putida was, in general, lower than that under glycolytic conditions (Fig. 5). The overall pattern was similar as observed before, and G6PDH-A seems to carry most of the flux, but higher activity was observed with NADP^+^ as cofactor. As the overall G6PDH levels were low under gluconeogenic growth conditions, the activity of other (potentially interfering) NADP^+^-specific dehydrogenases, e.g., Gnd (using the 6-phosphogluconate produced by G6PDH), becomes more pronounced; hence, the larger variability detected in these assays. Taken together, these observations led us to investigate if the presence and cofactor preference of multiple G6PDH isoforms could be a redox-adjusting mechanism in *Pseudomonas*.

### Genetically encoded cofactor specificity of G6PDH isozymes in P. putida KT2440.

**FIG 6:**
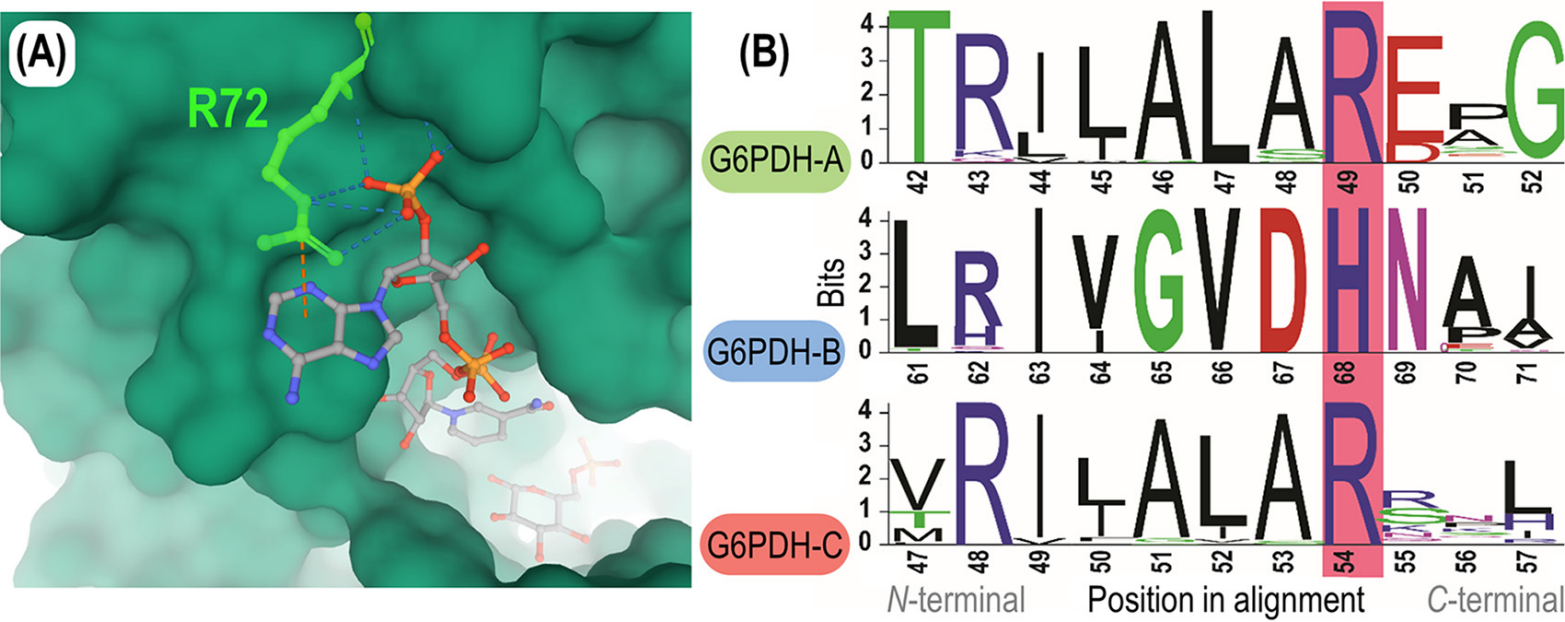
β2-α2 domain region of the G6PDH isozymes of P. putida KT2440. (A) Three-dimensional (3D) model of the cofactor binding pocket of the G6PDH enzyme of Trypanosoma cruzi. R72, the cofactor discriminating residue, located in the β2-α2 loop of the protein and corresponding to R49 in G6PDH-A of P. putida KT2440, is highlighted in green. R72 interacts with the 2′ phosphate group of NADP^+^ through four hydrogen bonds, as indicated with broken lines in the diagram. Crystal structure adapted from Mercaldi et al. (56). (B) Alignment of the amino acid sequence of the three G6PDH forms of P. putida KT2440. G6PDH-A, G6PDH-B, and G6PDH-C belong to family I, II, and III, respectively, of glucose-6-phosphate dehydrogenases. Orthologue entries from OrthoDB (61) were aligned for each family (*N *= 101, 66, and 50, respectively), and amino acid residues defining cofactor specificity (G6PDH-A^R49^, G6PDH-B^H68^, and G6PDH-C^R54^) are highlighted in pink.

An alignment of the amino acid sequences encoded by *zwfA*, *zwfB*, and *zwfC* (see Fig. S6) shows a relatively high degree of similarity: 54% between G6PDH-B and G6PDH-C, and 60% between G6PDH-A and G6PDH-C. No differential amino acid enrichment was evident among the isoforms—indicative, in some cases, of a role as a nutrient reserve (54). However, the information gathered so far points to distinct kinetic properties, and a predominant role of G6PDH-A under the experimental conditions tested. Hence, we searched for signatures in the dehydrogenase polypeptides that could point to differences in cofactor specificity. One elegant example in this direction is the analysis of the (unique) G6PDH enzyme of the parasitic euglenoid Trypanosoma cruzi (55). Mercaldi et al. (56) reported that this G6PDH displays a key amino acid residue in the β2-α2 domain, R72, which specifically interacts with the 2′-phosphate group of NADPH (Fig. 6A). Building on this rationale, this amino acid residue (arginine) was found to be determinant for the usage of NADP^+^ by G6PDHs from several organisms, including E. coli (57) and the lactic acid bacterium *L. mesenteroides* (58, 59). Furthermore, the G6PDH isozymes of Komagataeibacter hansenii, Burkholderia cepacia, and Pseudomonas fluorescens (biotype E), which are the most NAD^+^-specific G6PDHs characterized so far, lack this specific arginine residue (17, 21, 47, 57, 60). Thus, we reasoned that if a given G6PDH contains an arginine residue at the position predicted to interact with the 2′-phosphate moiety of NADP^+^, it can be considered to be either NADP^+^ or dual-cofactor specific, whereas other amino acids in this position would lower the NADP^+^ affinity of the enzyme.

10.1128/mSystems.00014-21.8 FIG S6 Alignment of the proteins encoded by *zwfA*, *zwfB*, and *zwfC* in P. putida KT2440. An “*” (asterisk) symbol indicates positions which have a single fully conserved residue. A “:” (colon) symbol indicates conservation between groups of strongly similar properties, and the “.” (period) mark indicates conservation between groups of weakly similar properties. Alignment was performed with CLUSTAL omega. Download FIG S6, PDF file, 0.7 MB. Copyright © 2021 Volke et al. 2021 Volke et al. https://creativecommons.org/licenses/by/4.0/ This content is distributed under the terms of the Creative Commons Attribution 4.0 International license.

Following this reasoning, three distinct groups—each highly conserved around the β2-α2 loop of the enzyme—were identified when all the known G6PDH orthologues across the entire *Pseudomonas* genus available in OrthoDB (61) were analyzed. Each of the G6PDH isoforms of P. putida KT2440 falls into one of these groups (Fig. 6B): two of these groups contain the residue arginine in the β2-α2 loop (i.e., G6PDH-A^R49^ and G6PDH-C^R54^), while one family contains a conserved histidine in the cofactor-discriminating position (i.e., G6PDH-B^H68^). These results are in agreement with the *in vitro* characterization of purified enzymes (Table 1) and the φ values reported for each variant, as the presence of an arginine residue at the cofactor-discriminating position of G6PDH-A and G6PDH-C correlates with increased affinity toward NADP^+^. G6PDH-B, in contrast, displays a histidine in this loop, which matches the lack of specificity of this variant toward the redox cofactor NADP^+^ (φ of G6PDH-B is ∼0.9) (Table 1). Taken together, these observations indicate that P. putida KT2440 harbors two main G6PDH functions relevant under *in vivo* conditions, G6PDH-A and G6PDH-B, and that these variants significantly differ in the cofactor specificity, probably enabling metabolic flexibility depending on the redox demand, which leads to the question of how widespread this correlation is across different bacterial species.

### The number and cofactor specificity of glucose-6-phosphate dehydrogenase isozymes correlate with the metabolic lifestyle across bacterial species.

**FIG 7:**
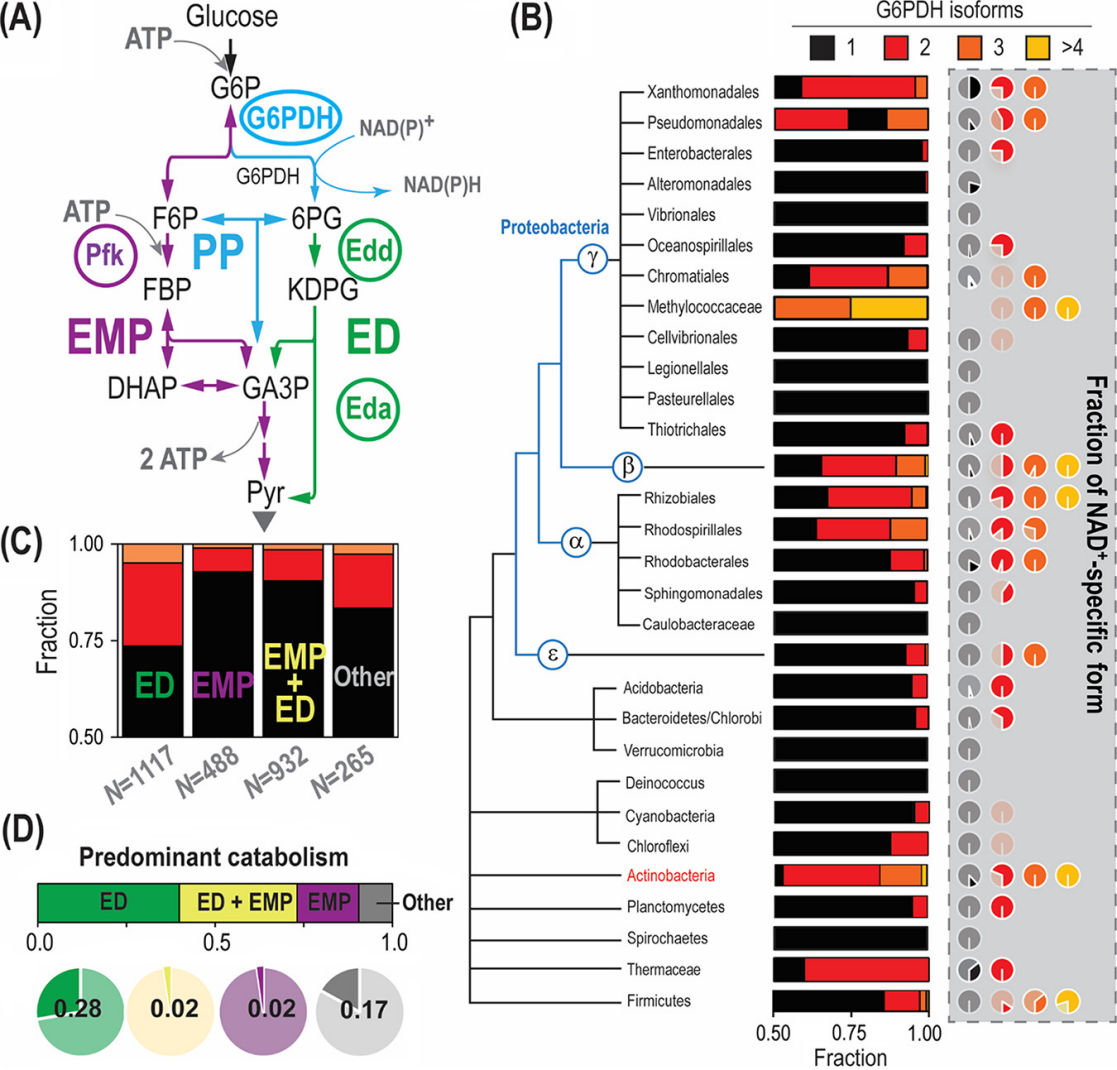
Cofactor specificity of G6PDH isoforms correlates with the metabolic lifestyle of the host. (A) Schematic comparison of the Entner-Doudoroff (ED) and Embden-Meyerhof-Parnas (EMP) glycolytic pathways. The EMP pathway converts glucose-6-phosphate (G6P) into fructose-6-phosphate (F6P), followed by a phosphorylation catalyzed by 6-phosphofructo-1-kinase (Pfk) that yields fructose-1,6-bisphosphate (FBP). Two molecules of glyceraldehyde-3-phosphate (GA3P) are finally formed through an aldolase and isomerization reaction. In the ED pathway, in contrast, G6P is oxidized to 6-phosphogluconate (6PG) through G6PDH (yielding a reducing equivalent) and 6-phosphogluconolactonase. After 6PG oxidation to 2-keto-3-deoxy-6-phosphogluconate (KDPG) by 6-phosphogluconate dehydratase (Edd), one GA3P molecule and one pyruvate (Pyr) molecule are formed by KDPG aldolase (Eda). Note that the EMP pathway yields twice the amount of ATP as the ED pathway. Color coding of different metabolic blocks and other abbreviations used herein are as indicated in the legend to Fig. 1. (B) Phylogenetic tree reconstruction of bacteria harboring different G6PDH variants. The fraction of microbial species in each order that harbor one, two, three, and four or more G6PDH isozymes is indicated together with the cofactor specificity of each of these fractions. NAD^+^ specificity was determined by the absence of the key arginine residue at the position interacting with the 2′-phosphate group of NADP^+^ (see Fig. 6B), and the fraction is indicated by a dark hue (a light shade is used for the NADP^+^-dependent variants). Information on *zwf* orthologues included in the analysis was gathered from OrthoDB (61). (C) Correlation between glycolytic pathways of the species analyzed and the number of *zwf* genes they harbor. The fraction of species relying solely on the ED pathway, both the ED and EMP pathways, or solely on the EMP pathway was established by assessing the presence of unique genes for each metabolic block. The cases where no genes of the ED or the EMP pathway could be found were categorized as “other.” The color code identifying the number of *zwf* orthologues is the same as for panel B. (D) Correlation between the cofactor preference of G6PDH isozymes and the catabolic lifestyle of the hosts. The fraction of NAD^+^-specific G6PDH forms is plotted according to the predominant glycolytic route (or their combination).

After identifying the presence of key residues defining NAD(P)^+^ acceptance in the G6PDH isozymes of strain KT2440, we were interested to explore if duplication of G6PDH-encoding genes is a widespread phenomenon in the bacterial domain and whether it has a connection with cofactor specificity. To this end, we searched all entries annotated as G6PDH in OrthoDB (61) and clustered them according to the bacterial metabolic lifestyle (as shown in the simplified Fig. 7A) and phylogenetic relationships between species (Fig. 7B). First, we observed that 88% of all analyzed *Pseudomonas* species harbor two or three G6PDH isozymes. Furthermore, we classified the corresponding isozymes as NADP^+^ or NAD^+^ dependent according to the presence or absence of the key arginine residue in the β2-α2 loop, respectively. While only 10% of the *Pseudomonas* species with a single *zwf* gene encode an NAD^+^-specific isoform, 65% of *Pseudomonas* with multiple *zwf* orthologues contain at least one NAD^+^-specific isozyme. Therefore, harboring multiple G6PDH isoforms with different cofactor specificities is an evolutionary trait common to the *Pseudomonas* genus. The analysis was subsequently broadened to the entire bacterial kingdom. Eukaryotes were excluded from this classification, as gene isoforms are known to be differentially expressed depending on the cell type or tissue (62). Archaea were likewise neglected, since archaeal G6PDH enzymes are not related to bacterial counterparts (63), which would make the cofactor specificity impossible to assign.

Many bacterial species were found to harbor multiple *zwf* genes (Fig. 7B), but these orthologues are not homogenously distributed throughout all bacterial orders. While some orders were observed to harbor a single G6PDH isoform almost exclusively (e.g., *Enterobacteriales* and *Alteromonadales*), around half of the species in other taxa, e.g., *Xanthomonadales* or *Actinobacteria*, were found to encode multiple G6PDH variants in their genomes. Within the proteobacterial phylum, only *Rhizobiales*, *Methylococcaceae*, and *Betaproteobacteria* contained ≥4 G6PDH isozymes (*Actinobacteria* and *Firmicutes* also shared this trait). To investigate the cofactor specificity of these G6PDHs, the amino acid sequences of all enzymes were aligned and analyzed according to the presence of the key residue discriminating NADP^+^. The number of organisms harboring an NAD^+^-specific G6PDH was very low if the corresponding species had a single copy of *zwf*, but the fraction increased drastically in bacteria with multiple G6PDH isoforms (Fig. 7B). The upsurge in the fraction of NAD^+^- to NADP^+^-preferring dehydrogenases was much higher than what would be expected just by chance. The general trend is that bacterial species seem to display an NAD^+^-specific isoform if they contain multiple G6PDH variants—while single G6PDH isoforms appear to be either NADP^+^ dependent or dual-cofactor specific. Given the heterogeneous distribution across bacterial orders, we wondered whether the multiplicity of G6PDH isoforms and their redox cofactor preference might correlate with the metabolic lifestyle of these species.

To investigate this possibility, all bacterial species were categorized according to the presence of signature glycolytic routes. According to the simplified classification that we implemented to this end, organisms harboring 6-phosphofructo-1-kinase (Pfk) were considered capable of running the EMP pathway, while 6-phosphogluconate dehydratase (Edd) or 2-keto-3-deoxy-6-phosphogluconate (KDPG) aldolase (Eda) was chosen as a marker of the ED pathway (Fig. 7A). If none of the genes that encoded these were found in a given species, the glycolytic strategy of the organism was labeled as “other”—a category that includes, among others, phosphoketolase-dependent and entirely fermentative lifestyles (1). We observed that the prevalence of multiple G6PDH variants strongly correlated with the use of the ED pathway as the sole glycolytic route in all orders (Fig. 7C), with the exception of the *Actinobacteria* phylum. If this taxon is excluded from the analysis, we observed that 26% of microbes relying on the ED pathway harbor multiple G6PDH isoforms, in contrast to just 7% and 10% for EMP- and EMP- and ED-utilizing organisms, respectively. Also, 17% of organisms lacking key enzymes of both the EMP and ED routes display multiple G6PDH isoforms. Therefore, the emerging picture is that bacteria that do not possess the capacity of running the linear EMP pathway are equipped with a larger fraction of NAD^+^-specific G6PDH variants. Furthermore, we confirmed that all glycolytic routes are well represented in this analysis (Fig. 7C and 7D), considering that the query for bacteria containing G6PDH enzyme(s) could potentially create a bias toward microbes using the ED route. A similar trend was observed for the cofactor specificity of the G6PDH isoforms (Fig. 7D): 28% and 17% of organisms using solely the ED pathway or neither the ED nor EMP routes, respectively, harbor a non-NADP^+^-specific G6PDH variant (i.e., NAD^+^ preferring). This observation contrasts with the percentage of bacteria relying either solely on the EMP pathway or on the EMP and ED routes that carry an NAD^+^-specific G6PDH variant (2% for each of them).

A clear outlier in this analysis are members of *Actinobacteria* (Fig. 7B), and they deserve a separate discussion. The majority of organisms in the *Actinobacteria* phylum often contain the necessary genes for running both the EMP and ED pathways. Therefore, a relatively low abundance of organisms with multiple isoenzymes and NAD^+^-dependent variants would have been expected according to the interpretation above. However, we observed that almost half of the species display multiple *zwf* genes, and NADH-yielding isozymes are relatively abundant. Accordingly, *Actinobacteria* are only very distantly related to the other taxa analyzed in this study, and they might have evolved different metabolic strategies for carbon and redox balancing. It appears that the EMP pathway is preferred over the ED and PP pathways for glucose catabolism in this phylum. Streptomyces coelicolor, for instance, diverts around half of the glycolytic flux through the EMP route and the remaining half through the ED pathway (64). Similarly, 62% and 52% of the glucose uptake flux is directed through the reaction catalyzed by the G6PDHs in Corynebacterium glutamicum and Streptomyces lividans, respectively (65, 66). *Nonomuraea* sp. strain ATCC 39727 even uses the ED pathway predominantly, although the genes encoding enzymes of the EMP pathway are present in the genome (67). Thus, the high flux through G6PDH—even when the EMP pathway is present—explains the high abundance of NADH-yielding isoforms in this phylum.

## DISCUSSION

The notion that enzymes became highly specialized to execute a single function is often misleading—many of them exhibit a wide spectrum of substrates leading to accuracy-rate tradeoffs, thereby affecting evolutionary trajectories (68). We examined this issue in the model environmental bacterium P. putida, and we found that the three G6PDH isozymes of strain KT2440 exhibit different cofactor specificities. While G6PDH-A (the production of which is induced by glucose) uses NADP^+^ and NAD^+^ at similar proportions, G6PDH-B (which is constitutively expressed) prefers NAD^+^ as the cofactor. According to the physiological characterization of mutants lacking *zwfA*, this variant displays the most prominent role in batch cultures with glucose as the carbon source, where substrate abundance in the medium is high and cytoplasmic G6P concentrations are typically elevated (24, 69). This situation helps explain the higher *K_m_* of G6PDH-A for G6P than of the other two variants and hints to an important contribution of G6PDH-B to catabolism during carbon limitation. The interplay between the activities afforded by G6PDH-A and G6PDH-B appears to balance NADPH production on different carbon sources, since only a small fraction of the carbon flux is directed through G6PDH during glucose-dependent growth, whereas almost the entire flux is funneled through this reaction when the cells are grown on fructose or ribose.

G6PDH-C, in contrast, has extremely low turnover both *in vivo* and *in vitro*, and its deletion had negligible effects on bacterial growth. Thus, the function of this isozyme remains elusive, and its role could even be structural or regulatory rather than metabolic. Examples of this sort (where dehydrogenase enzymes display a nonmetabolic function) include one of the GA3P dehydrogenase isozymes of the pathogen Neisseria meningitidis which plays an important role in the adhesion of the bacterium to host cells (70). Another instance is the structural role of lactate dehydrogenase in the lenses of crocodiles and birds (71). While it is possible that G6PDH-C requires a protein(s) and/or allosteric effector(s) absent in our enzymatic assays to be rendered fully functional, this is usually not the case for this type of dehydrogenase activity. Another possibility is that this enzyme prefers another sugar substrate different from G6P, as demonstrated for the sulfoquinovose catabolic pathway of P. putida SQ1 (72), or an alternative redox cofactor besides NAD(P)^+^. These scenarios notwithstanding, we hypothesize that the existence of G6PDH-A and G6PDH-B as the main metabolism-related G6PDH isozymes “freed” G6PDH-C to explore new functions. This phenomenon (also known as “moonlighting”) is a well-known feature of catabolic modules in environmental bacteria which acquired the capability of processing alternative substrates via gene duplication and enzyme specialization, usually in connection with a local transcriptional factor (73, 74), as observed for the gene encoding the RpiR-like regulator and *zwfC*. From a more general perspective, the so-called “patchwork” model theorizes that primitive enzymes were highly promiscuous to confer a larger degree of catalytic versatility when the pool of available biocatalysts was limited (75, 76). Besides the rich panoply of biochemical reactions, P. putida may bear underground metabolic pathways as a basis of its remarkable capacity to adapt to changing environments (77).

One way or the other, it seems that G6PDH-A and G6PDH-B carry most of the G6PDH activity in this bacterial species. The use of multiple such isoenzymes is prevalent in organisms lacking a functional EMP pathway and correlates with the use of NAD^+^-specific variants. We argue that this occurrence confers metabolic flexibility to the host without altering carbon fluxes. The dual cofactor specificity of G6PDH-B probably balances redox cofactor production in a narrow window just by demand, as the NADPH/NADH ratio associated with G6PDH activity is highly dependent on substrate (i.e., NADP^+^ and NAD^+^) availability (22). An increase in the demand of a specific cofactor leads to a higher concentration of its oxidized form, thereby shifting substrate availability and, subsequently, the cofactor output. The two active isozymes warrant a broader window for cofactor balancing to P. putida, and may provide a reserve flux capacity under oxidative stress. Both G6PDH-A and G6PDH-B are present in the cells simultaneously under all the experimental conditions investigated herein. G6PDH-A is inhibited by NADPH, with a *K_i_* (NADPH) of 112 μM, lower than the intracellular NADPH concentration of 276 μM observed in strain KT2440 when grown under nonstressed conditions (78). A higher flux is needed to compensate for this inhibition, and if G6PDH-B supplies the extra activity, NADH would be produced. Under oxidative stress, the concentration of NADPH decreases and releases the inhibition on G6PDH-A, thus increasing the flux through this node and thereby producing NADPH. Furthermore, the different metabolic specialization of the two variants is also reflected by the lower *K_m_* of G6PDH-B for G6P than G6PDH-A (around 5-fold), thus favoring G6P processing through G6PDH-B in metabolic states with low substrate levels, e.g., growth on gluconeogenic carbon sources.

From a broader perspective, the correlation between the predominant glycolytic strategy of a given microorganism and the presence of G6PDH isozymes displaying different cofactor specificities seems to be largely dictated by the need of balancing the redox status on different carbon sources according to environmental conditions. As indicated above, bacteria relying on the EMP pathway for sugar catabolism can increase flux into the PP or the ED pathway through G6PDH whenever an extra supply of NADPH is needed. This is actually the case under oxidative stress conditions, as previously shown in E. coli, yeast, and mammalian cells (11, 79, 80). Such a strategy is obviously not possible in microbes solely relying on the ED pathway, and the use of isozymes with different cofactor specificities becomes more prominent. Other redox-adjusting mechanisms include the use of peripheral (oxidative) pathways in the first steps of sugar processing, e.g., the gluconate/2-ketogluconate loop of P. putida (Fig. 1), together with allosteric control of enzyme activities at key metabolic steps. Only 20% of the glucose is directly phosphorylated to G6P in P. putida (before being channeled through G6PDH), whereas 80% of the sugar is oxidized in the gluconate/2-ketogluconate loop and thus bypasses G6PDH (24). Accordingly, P. putida strongly expresses *zwfA*, encoding the NADPH-forming variant, under this condition, thereby adjusting the NADPH output by tuning the flux split between glucose phosphorylation and oxidation. Besides, the metabolism of glucose generates 24 reducing equivalents per molecule, while gluconate yields 22 reducing equivalents (81); thus, the conversion of glucose to Pyr requires one additional oxidation step in comparison to that for gluconate. This adds a further layer of transcriptional control on the *zwf* orthologues of P. putida: expression of *zwfA* is enhanced in the presence of glucose to ensure this additional oxidation step, which is not required for the oxidation of gluconate. Given the absence of any overflow metabolism, this bacterium achieves redox balancing through the activity of alternative dehydrogenases rather than by producing fermentation products. These observations will also open up new strategies toward engineering the rich metabolism of this species by harnessing the wealth of enzymatic activities typically found in *Pseudomonas* (82–84).

## MATERIALS AND METHODS

### Bacterial strains, plasmids, and culture conditions.

Chemicals were supplied by Sigma-Aldrich Co. (St. Louis, MO, USA) if not otherwise stated. All bacterial strains used in this study are listed in Table S1 in the supplemental material. Cultures of P. putida KT2440 and E. coli as well as their derivatives were incubated at 30°C and 37°C, respectively. For standard applications, routine cloning procedures, and during genome engineering manipulations, cells were grown in LB medium (containing 10 g·liter^−1^ tryptone, 5 g·liter^−1^ yeast extract, and 10 g·liter^−1^ NaCl, pH 7.0). All liquid cultures were agitated at 250 rpm (E. coli) or 180 rpm (P. putida) in an orbital MaxQ 8000 shaker incubator (Thermo Fisher Scientific, Waltham, MA, USA). Solid medium contained 15 g·liter^−1^ agar. Kanamycin (Km) and gentamicin (Gm) were added whenever needed to retain plasmids at 50 μg·ml^−1^ and 10 μg·ml^−1^, respectively. M9 minimal medium (24) supplemented with 20 mM citrate, 20 mM glucose, 24 mM ribose, 20 mM fructose, 40 mM glycerol, or 20 mM gluconate was used for phenotypic characterization of P. putida KT2440 (note that the total amount of carbon atoms was kept constant across experimental conditions). Growth rates were determined by fitting the temporal changes in optical density measured at 600 nm (OD_600_) to the exponential growth model. Optical densities were recorded in a microplate reader (Elx808; BioTek Instruments, Winooski, VT, USA).

For the assessment of relative fluorescence intensities using reporter constructs, cultures were grown in M9 medium supplemented with the corresponding carbon source overnight. These cultures were used to inoculate wells of a 96-well plate containing the same medium. The cultures were shaken at 30°C in a Synergy HI plate reader (BioTek Instruments), and growth and fluorescence were followed by spectrophotometry at 600 nm and 500/530 nm (excitation/emission wavelength), respectively, essentially as described before (85). The highest relative fluorescence intensity is reported, corresponding to the onset of the stationary phase.

### General cloning procedures and plasmid and mutant construction.

All oligonucleotides and plasmids used in this work are listed in Table S2 and Table S1, respectively. Phusion Hot Start II high-fidelity DNA polymerases (Thermo Fisher Scientific, Waltham, MA, USA) was used for DNA amplification according to the manufacturer’s specifications. For colony PCR, the commercial One*Taq* master mix (New England BioLabs, Ipswich, MA, USA) was used according to the manufacturer’s instructions. E. coli DH5α was used for general cloning purposes, while E. coli DH5α λ*pir* was employed when cloning and maintaining replicons with the conditional, Π-dependent origin of replication *RK6* (Table S1). Chemically competent E. coli cells were prepared and transformed with plasmids according to well-established protocols (86). P. putida was rendered electrocompetent by washing the biomass from saturated (24 h) LB medium cultures with 0.3 M sucrose, and cells were routinely transformed with plasmids by electroporation according to the protocol of Choi et al. (87).

10.1128/mSystems.00014-21.2 TABLE S2 Oligonucleotides used in this study. Download Table S2, PDF file, 0.4 MB. Copyright © 2021 Volke et al. 2021 Volke et al. https://creativecommons.org/licenses/by/4.0/ This content is distributed under the terms of the Creative Commons Attribution 4.0 International license.

Genomic DNA was extracted from P. putida KT2440 using the DNeasy blood and tissue kit (Qiagen). Plasmids for gene deletion were constructed by amplification of 500-bp-long fragments up- and downstream of the corresponding gene. These fragments were spliced by overlap extension PCR and ligated into the digested backbones. USER cloning was performed according to Volke et al. (28). Similarly, the plasmids containing the promoter regions of *zwf* and *zwfA* were constructed by amplifying the corresponding genomic regions with the primer pairs P*_zwfA_*_EcoRI-F/P*_zwfA_*_BamHI-R and P*_zwfB_*_EcoRI-F/P*_zwfB_*_BamHI-R (Table S2), respectively, and digesting the fragments and vector pSEVA237Y with EcoRI and BamHI, followed by ligation. Integrity of all constructs was checked by DNA sequencing. Genomic DNA was employed as the template for the amplification of *zwfB* and *zwfC* with the primer pairs Zwf_NdeI-F/Zwf_BamHI-R and ZwfB_NdeI-F/ZwfB_BamHI-R (Table S2), respectively. Plasmid pET28a and the amplification products were restricted with NdeI and BamHI, separated on a 1% (wt/vol) agarose gel, and ligated. Ligation was accomplished using T4 DNA ligase (New England BioLabs) according to the protocol provided by the manufacturer and giving rise to expression plasmids pET28a·*zwfB^Pp^* and pET28a·*zwfC^Pp^*. Then, electrocompetent E. coli DH5α cells were transformed with these ligation products. Positive clones, identified by colony PCR, were verified by DNA sequencing.

### Determination of G6PDH activities in cell extracts.

A 10-ml aliquot of M9 medium supplemented with the corresponding carbon source was inoculated with P. putida KT2440 cells from a single colony and incubated for 16 h. This culture was used to inoculate a 100-ml Erlenmeyer flask containing 20 ml of fresh medium to achieve an initial OD_600_ of 0.05. The culture was grown in an orbital shaker (180 rpm) to an OD_600_ of 0.5. Cells were then harvested (4,000 × *g*, 10 min, 4°C), the supernatant was discarded, and the pellet was placed in an ice bath. The pellet was suspended in 0.5 ml of buffer A, containing 50 mM Tris-HCl, 5 mM NaCl, 5 mM MgCl_2_, and 5% (vol/vol) glycerol, pH 8.0. Cells were mixed with 0.3 g of glass beads (212 to 300 μm, acid washed, Sigma-Aldrich Co. G1277) and disrupted at 6,000 rpm for 20 s (Precellys 24; Berlin Technology, Berlin, Germany). Unbroken cells and cell debris were pelleted by centrifugation at 17,000 × *g* for 2 min at 4°C. The supernatant was transferred to a new tube. Protein concentration of the supernatant was determined by means of the Bradford assay (88) using a commercial kit (Pierce, Thermo Fisher). For these measurements, 5 μg of total protein, 0.25 mM NAD(P)^+^ (as indicated in the text), and 2 mM G6P were mixed in 0.2 ml of buffer A and incubated at 30°C. All stock solutions were freshly prepared. The initial rate of formation of NAD(P)H was followed by spectrophotometry at 340 nm in a Synergy HI plate reader (BioTek Instruments).

### Enzyme production and purification and kinetic assays.

E. coli BL21(DE3) transformed either with plasmid pET28a·*zwfB* or pET28a·*zwfC* was grown in LB medium at 37°C with agitation until OD_600_ of ≈0.5. At that point, isopropyl-β-d-1-thiogalactopyranoside (IPTG) was added to the cultures, and the temperature of the incubator was decreased to 25°C. Cells were incubated for 16 to 20 h under these conditions. Cells from these IPTG-induced cultures were harvested by centrifugation (5,000 × *g*, 4°C, 30 min), washed twice with ice-cold buffer A, and resuspended in buffer A supplemented with 2 mM (l+d)-dithiothreitol, additional NaCl (up to 100 mM), 20 mM imidazole, and a protease inhibitor cocktail (Roche) prepared as recommended by the manufacturer. Resuspended cells were sonicated on ice and ultracentrifuged (30,000 × *g*, 4°C, 60 min). The supernatants obtained after this step were loaded in 5-ml His-trap columns (GE Healthcare Systems, Chicago, IL, USA) pre-equilibrated with buffer A supplemented with 100 mM NaCl and 20 mM imidazole. His-tagged proteins were eluted by gradually increasing the concentration of imidazole in the buffer flowing through the His-trap columns. The gradients of imidazole-containing buffer were linear (from 20 mM to 500 mM), corresponding to 40 times the His-trap column volume. Eluted fractions (1.5 ml) were collected, and the G6PDH activity was measured by following the formation of NADPH by spectrophotometry at 340 nm as explained in the previous section. Fractions containing G6PDH activity levels in the upper quintile of all samples were pooled, concentrated, and equilibrated in buffer A. Purity of G6PDH-B and G6PDH-C was evaluated by separation by SDS-PAGE. Pure proteins were preserved with 50% (vol/vol) glycerol, added gradually to avoid precipitation of the purified enzyme preparation, and protein stocks were stored at −20°C until they were employed for kinetic assays.

Samples of pure protein were equilibrated in buffer A at 4°C before the kinetic assays. The specific activities of samples were compared with the values registered before the storage, and no significant decline in the activities was observed after 2 weeks of storage under the conditions mentioned above. The stocks of G6P, NAD^+^, and NADP^+^ were neutralized, and their concentrations were determined by titration as described by Olavarría et al. (48). The kinetic assays were performed in buffer A, at 30°C, supplemented with NAD(P)^+^ and G6P at different concentrations as indicated in the text. A UV-visible (UV/Vis) Synergy 2 spectrophotometer (BioTek Instruments) and nonbinding flat-bottom 96-wells plates (model 655901; Greiner Bio-One GmbH, Kremsmünster, Austria) were used for recording spectrophotometric changes in the reaction mixtures. Separate assays were performed to determine the optimal concentration of enzyme to minimize inactivation (see Fig. S7) and to obtain initial conversion rate (*V*_0_) estimations before consumption of 5% of the initial amount of substrate (89). Such preliminary assays were also employed to obtain *a priori* estimations of *K_m_* values using the method of linear direct plotting (90). Initial rates were assessed with NAD(P)^+^ concentrations ranging from 20 to 2,000 μM with different fixed concentrations of G6P, ranging from 95 to 5,000 μM (Fig. S3). Kinetic parameters obtained through the global fitting procedure were employed to estimate the relative formation of NADH and NADPH in the reactions catalyzed by G6PDH-B and G6PDH-C (22). The DYNAFIT software package (91) was used for this analysis. It allows for global fittings, i.e., using data obtained using different concentrations of substrate, enzyme, and/or modifiers simultaneously.

10.1128/mSystems.00014-21.9 FIG S7 Selwyn plot of three representative reaction progress curves obtained with G6PDH-B at three different enzyme concentrations. Overlapping of the plots suggested *a priori* that no enzyme inactivation occurred. Further model discrimination analysis with DYNAFIT comparing product competitive inhibition models, with and without enzyme degradation, endorsed this null hypothesis. The reactions were studied in buffer A (50 mM Tris-HCl, 5 mM NaCl, 5 mM MgCl2, 5% [vol/vol] glycerol, pH 8.0), supplemented with 200 μM NADP^+^ and 4 mM G6P. Reactions were followed over 30 min, and for the sake of simplicity, other curves and data points are not shown. Download FIG S7, PDF file, 0.6 MB. Copyright © 2021 Volke et al. 2021 Volke et al. https://creativecommons.org/licenses/by/4.0/ This content is distributed under the terms of the Creative Commons Attribution 4.0 International license.

### Phylogenetic analysis.

Amino acid sequences of proteins with annotated G6PDH function were acquired from the orthologue database OrthoDB (61). Sequences of each taxonomic order were aligned (92) and categorized according to the key catalytic residue corresponding to R50 in E. coli (57). Proteins containing an arginine residue in the corresponding module of G6PDH were considered to be NADPH specific, while all other amino acids in this position were considered to have a loosened cofactor discrimination. In the following step, organisms carrying these variants were categorized as using (i) the ED pathway, if their genomes encoded the signature enzyme KDPG aldolase, or (ii) the EMP pathway, if they contained a 6-phosphofructo-1-kinase annotated in the OrthoDB. Note that both KDPG aldolase and 6-phosphofructo-1-kinase are unique for each type of metabolic pathway (2). If none of these proteins were found, the predominant metabolism of the corresponding host was categorized as “unknown.” The phylogenetic tree, blending all these constraints, was drawn according to Jun et al. (93), and sequence logos were generated with WebLogo3 (94).

### Data and statistical analysis.

Initial rates obtained in experiments with purified enzymes were globally fitted using the DYNAFIT software version 4 (Biokin Ltd., Watertown, MA, USA) (91). Beyond the estimation of the best-fitted values for each kinetic parameter, this software enabled a model discrimination analysis among different reaction mechanisms to determine which of them best explained the observed results. Due to the nonlinear relationship between rates and substrate concentrations, the uncertainty in the estimations of the kinetic parameters was expressed as 95% confidence intervals. All other experiments reported in this study were independently repeated at least in biological triplicates (as indicated in the corresponding figure or table legend), and the mean value of the corresponding parameter ± standard deviation is presented. Whenever relevant, the level of significance of the statistical differences was evaluated by means of the two-tailed, homoscedastic Student’s *t* test, with α of 0.01 (**) or α of 0.05 (*), as indicated in the respective figure legends.

### Data availability.

All raw data and materials will be made available upon request.

## Supplementary Material

Reviewer comments

## References

[1] Folch PL, Bisschops MMM, Weusthuis RA. 13 January 2021. Metabolic energy conservation for fermentative product formation. Microb Biotechnol doi:10.1111/1751-7915.13746. PMC8085960 33438829

[2] Flamholz A, Noor E, Bar-Even A, Liebermeister W, Milo R. 2013. Glycolytic strategy as a tradeoff between energy yield and protein cost. Proc Natl Acad Sci U S A 110:10039–10044. doi:10.1073/pnas.1215283110. 23630264 PMC3683749

[3] Conway T. 1992. The Entner-Doudoroff pathway: history, physiology and molecular biology. FEMS Microbiol Rev 9:1–27. doi:10.1111/j.1574-6968.1992.tb05822.x. 1389313

[4] Fuhrer T, Fischer E, Sauer U. 2005. Experimental identification and quantification of glucose metabolism in seven bacterial species. J Bacteriol 187:1581–1590. doi:10.1128/JB.187.5.1581-1590.2005. 15716428 PMC1064017

[5] Schada von Borzyskowski L, Bernhardsgrütter I, Erb TJ. 2020. Biochemical unity revisited: microbial central carbon metabolism holds new discoveries, multi-tasking pathways, and redundancies with a reason. Biol Chem 401:1429–1441. doi:10.1515/hsz-2020-0214. 32990641

[6] Kopp D, Sunna A. 2020. Alternative carbohydrate pathways—enzymes, functions and engineering. Crit Rev Biotechnol 40:895–912. doi:10.1080/07388551.2020.1785386. 32654530

[7] Pollak N, Dölle C, Ziegler M. 2007. The power to reduce: pyridine nucleotides—small molecules with a multitude of functions. Biochem J 402:205–218. doi:10.1042/BJ20061638. 17295611 PMC1798440

[8] Nordberg J, Arnér ES. 2001. Reactive oxygen species, antioxidants, and the mammalian thioredoxin system. Free Radic Biol Med 31:1287–1312. doi:10.1016/S0891-5849(01)00724-9. 11728801

[9] Arnér ES, Holmgren A. 2000. Physiological functions of thioredoxin and thioredoxin reductase. Eur J Biochem 267:6102–6109. doi:10.1046/j.1432-1327.2000.01701.x. 11012661

[10] Fuhrer T, Sauer U. 2009. Different biochemical mechanisms ensure network-wide balancing of reducing equivalents in microbial metabolism. J Bacteriol 191:2112–2121. doi:10.1128/JB.01523-08. 19181802 PMC2655529

[11] Christodoulou D, Link H, Fuhrer T, Kochanowski K, Gerosa L, Sauer U. 2018. Reserve flux capacity in the pentose phosphate pathway enables *Escherichia coli*'s rapid response to oxidative stress. Cell Syst 6:569–578. doi:10.1016/j.cels.2018.04.009. 29753645

[12] Sandoval JM, Arenas FA, Vásquez CC. 2011. Glucose-6-phosphate dehydrogenase protects *Escherichia coli* from tellurite-mediated oxidative stress. PLoS One 6:e25573. doi:10.1371/journal.pone.0025573. 21984934 PMC3184162

[13] Lundberg BE, Wolf RE, Dinauer MC, Xu Y, Fang FC. 1999. Glucose 6-phosphate dehydrogenase is required for *Salmonella* Typhimurium virulence and resistance to reactive oxygen and nitrogen intermediates. Infect Immun 67:436–438. doi:10.1128/IAI.67.1.436-438.1999. 9864251 PMC96332

[14] Ragunathan S, Levy HR. 1994. Purification and characterization of the NAD-preferring glucose 6-phosphate dehydrogenase from *Acetobacter hansenii* (*Acetobacter xylinum*). Arch Biochem Biophys 310:360–366. doi:10.1006/abbi.1994.1179. 8179320

[15] Anderson BM, Anderson CD. 1995. Purification and characterization of *Azotobacter vinelandii* glucose-6-phosphate dehydrogenase: dual coenzyme specificity. Arch Biochem Biophys 321:94–100. doi:10.1006/abbi.1995.1372. 7639541

[16] Canonaco F, Hess TA, Heri S, Wang T, Szyperski T, Sauer U. 2001. Metabolic flux response to phosphoglucose isomerase knock-out in *Escherichia coli* and impact of overexpression of the soluble transhydrogenase UdhA. FEMS Microbiol Lett 204:247–252. doi:10.1111/j.1574-6968.2001.tb10892.x. 11731130

[17] Olavarría K, de Ingeniis J, Zielinski DC, Fuentealba M, Muñoz R, McCloskey D, Feist AM, Cabrera R. 2014. Metabolic impact of an NADH-producing glucose-6-phosphate dehydrogenase in *Escherichia coli*. Microbiology 160:2780–2793. doi:10.1099/mic.0.082180-0. 25246670

[18] Klingner A, Bartsch A, Dogs M, Wagner-Döbler I, Jahn D, Simon M, Brinkhoff T, Becker J, Wittmann C. 2015. Large-scale ^13^C flux profiling reveals conservation of the Entner-Doudoroff pathway as a glycolytic strategy among marine bacteria that use glucose. Appl Environ Microbiol 81:2408–2422. doi:10.1128/AEM.03157-14. 25616803 PMC4357956

[19] Chavarría M, Nikel PI, Pérez-Pantoja D, de Lorenzo V. 2013. The Entner-Doudoroff pathway empowers *Pseudomonas putida* KT2440 with a high tolerance to oxidative stress. Environ Microbiol 15:1772–1785. doi:10.1111/1462-2920.12069. 23301697

[20] Lessie TG, Wyk JC. 1972. Multiple forms of *Pseudomonas multivorans* glucose-6-phosphate and 6-phosphogluconate dehydrogenases: differences in size, pyridine nucleotide specificity, and susceptibility to inhibition by adenosine 5'-triphosphate. J Bacteriol 110:1107–1117. doi:10.1128/JB.110.3.1107-1117.1972. 4402279 PMC247534

[21] Lessmann D, Schimz KL, Kurz G. 1975. d-Glucose-6-phosphate dehydrogenase (Entner-Doudoroff enzyme) from *Pseudomonas fluorescens*. Purification, properties and regulation. Eur J Biochem 59:545–559. doi:10.1111/j.1432-1033.1975.tb02481.x. 1257

[22] Olavarría K, Pupke Marone M, da Costa Oliveira H, Roncallo JC, Nogales da Costa Vasconcelos F, Ferreira da Silva L, Cabrera Gómez JG. 2015. Quantifying NAD(P)H production in the upper Entner-Doudoroff pathway from *Pseudomonas putida* KT2440. FEBS Open Bio 5:908–915. doi:10.1016/j.fob.2015.11.002. PMC4669411 26702395

[23] DangThu Q, Jang SH, Lee C. 2020. Biochemical comparison of two glucose 6-phosphate dehydrogenase isozymes from a cold-adapted *Pseudomonas mandelii*. Extremophiles 24:501–509. doi:10.1007/s00792-020-01171-3. 32346763

[24] Nikel PI, Chavarría M, Fuhrer T, Sauer U, de Lorenzo V. 2015. *Pseudomonas putida* KT2440 strain metabolizes glucose through a cycle formed by enzymes of the Entner-Doudoroff, Embden-Meyerhof-Parnas, and pentose phosphate pathways. J Biol Chem 290:25920–25932. doi:10.1074/jbc.M115.687749. 26350459 PMC4646247

[25] Nikel PI, Fuhrer T, Chavarría M, Sánchez-Pascuala A, Sauer U, de Lorenzo V. 11 January 2021. Reconfiguration of metabolic fluxes in *Pseudomonas putida* as a response to sub-lethal oxidative stress. ISME J doi:10.1038/s41396-020-00884-9. PMC8163872 33432138

[26] Kohlstedt M, Wittmann C. 2019. GC-MS-based ^13^C metabolic flux analysis resolves the parallel and cyclic glucose metabolism of *Pseudomonas putida* KT2440 and *Pseudomonas aeruginosa* PAO1. Metab Eng 54:35–53. doi:10.1016/j.ymben.2019.01.008. 30831266

[27] Kim J, Jeon CO, Park W. 2008. Dual regulation of *zwf-1* by both 2-keto-3-deoxy-6-phosphogluconate and oxidative stress in *Pseudomonas putida*. Microbiology 154:3905–3916. doi:10.1099/mic.0.2008/020362-0. 19047757

[28] Volke DC, Friis L, Wirth NT, Turlin J, Nikel PI. 2020. Synthetic control of plasmid replication enables target- and self-curing of vectors and expedites genome engineering of *Pseudomonas putida*. Metab Eng Commun 10:e00126. doi:10.1016/j.mec.2020.e00126. 32215253 PMC7090339

[29] Wirth NT, Kozaeva E, Nikel PI. 2020. Accelerated genome engineering of *Pseudomonas putida* by I-SceI―mediated recombination and CRISPR-Cas9 counterselection. Microb Biotechnol 13:233–249. doi:10.1111/1751-7915.13396. 30861315 PMC6922521

[30] Chavarría M, Kleijn RJ, Sauer U, Pflüger-Grau K, de Lorenzo V. 2012. Regulatory tasks of the phosphoenolpyruvate-phosphotransferase system of *Pseudomonas putida* in central carbon metabolism. mBio 3:e00028-12. doi:10.1128/mBio.00028-12. 22434849 PMC3312210

[31] Wilkes RA, Mendonca CM, Aristilde L. 2019. A cyclic metabolic network in *Pseudomonas protegens* Pf-5 prioritizes the Entner-Doudoroff pathway and exhibits substrate hierarchy during carbohydrate co-utilization. Appl Environ Microbiol 85:e02084-18. doi:10.1128/aem.02084-18. 30366991 PMC6293094

[32] del Castillo T, Duque E, Ramos JL. 2008. A set of activators and repressors control peripheral glucose pathways in *Pseudomonas putida* to yield a common central intermediate. J Bacteriol 190:2331–2339. doi:10.1128/JB.01726-07. 18245293 PMC2293218

[33] Nikel PI, Kim J, de Lorenzo V. 2014. Metabolic and regulatory rearrangements underlying glycerol metabolism in *Pseudomonas putida* KT2440. Environ Microbiol 16:239–254. doi:10.1111/1462-2920.12224. 23967821

[34] Poblete-Castro I, Wittmann C, Nikel PI. 2020. Biochemistry, genetics, and biotechnology of glycerol utilization in *Pseudomonas* species. Microb Biotechnol 13:32–53. doi:10.1111/1751-7915.13400. 30883020 PMC6922529

[35] D'Arrigo I, Cardoso JGR, Rennig M, Sonnenschein N, Herrgård MJ, Long KS. 2019. Analysis of *Pseudomonas putida* growth on non-trivial carbon sources using transcriptomics and genome-scale modelling. Environ Microbiol Rep 11:87–97. doi:10.1111/1758-2229.12704. 30298597

[36] Beckers V, Poblete-Castro I, Tomasch J, Wittmann C. 2016. Integrated analysis of gene expression and metabolic fluxes in PHA-producing *Pseudomonas putida* grown on glycerol. Microb Cell Fact 15:73. doi:10.1186/s12934-016-0470-2. 27142075 PMC4855977

[37] Vicente M, Pedro MA, Torrontegui G, Cánovas JL. 1975. The uptake of glucose and gluconate by *Pseudomonas putida*. Mol Cell Biochem 7:59–64. doi:10.1007/BF01732164. 1134500

[38] Chavarría M, Goñi-Moreno A, de Lorenzo V, Nikel PI. 2016. A metabolic widget adjusts the phosphoenolpyruvate-dependent fructose influx in *Pseudomonas putida*. mSystems 1:e00154-16. doi:10.1128/mSystems.00154-16. 27933319 PMC5141268

[39] Sawyer MH, Baumann P, Baumann L, Berman SM, Cánovas JL, Berman RH. 1977. Pathways of d-fructose catabolism in species of *Pseudomonas*. Arch Microbiol 112:49–55. doi:10.1007/BF00446653. 139135

[40] Phibbs PV, McCowen SM, Feary TW, Blevins WT. 1978. Mannitol and fructose catabolic pathways of *Pseudomonas aeruginosa* carbohydrate-negative mutants and pleiotropic effects of certain enzyme deficiencies. J Bacteriol 133:717–728. doi:10.1128/JB.133.2.717-728.1978. 146701 PMC222080

[41] Sudarsan S, Dethlefsen S, Blank LM, Siemann-Herzberg M, Schmid A. 2014. The functional structure of central carbon metabolism in *Pseudomonas putida* KT2440. Appl Environ Microbiol 80:5292–5303. doi:10.1128/AEM.01643-14. 24951791 PMC4136102

[42] Alberty RA. 2001. Systems of biochemical reactions from the point of view of a semigrand partition function. Biophys Chem 93:1–10. doi:10.1016/S0301-4622(01)00202-2. 11604212

[43] Bennett BD, Kimball EH, Gao M, Osterhout R, Van Dien SJ, Rabinowitz JD. 2009. Absolute metabolite concentrations and implied enzyme active site occupancy in *Escherichia coli*. Nat Chem Biol 5:593–599. doi:10.1038/nchembio.186. 19561621 PMC2754216

[44] Maleki S, Mærk M, Hrudikova R, Valla S, Ertesvåg H. 2017. New insights into *Pseudomonas fluorescens* alginate biosynthesis relevant for the establishment of an efficient production process for microbial alginates. N Biotechnol 37:2–8. doi:10.1016/j.nbt.2016.08.005. 27593394

[45] Lessie T, Neidhardt FC. 1967. Adenosine triphosphate-linked control of *Pseudomonas aeruginosa* glucose-6-phosphate dehydrogenase. J Bacteriol 93:1337–1345. doi:10.1128/JB.93.4.1337-1345.1967. 4382249 PMC276606

[46] O'Brien RW. 1975. Enzymatic analysis of the pathways of glucose catabolism and gluconeogenesis in *Pseudomonas citronellolis*. Arch Microbiol 103:71–76. doi:10.1007/BF00436332. 239656

[47] Maleki S, Mærk M, Valla S, Ertesvåg H. 2015. Mutational analyses of glucose dehydrogenase and glucose-6-phosphate dehydrogenase genes in *Pseudomonas fluorescens* reveal their effects on growth and alginate production. Appl Environ Microbiol 81:3349–3356. doi:10.1128/AEM.03653-14. 25746989 PMC4407215

[48] Olavarría K, Valdés D, Cabrera R. 2012. The cofactor preference of glucose-6-phosphate dehydrogenase from *Escherichia coli*—modeling the physiological production of reduced cofactors. FEBS J 279:2296–2309. doi:10.1111/j.1742-4658.2012.08610.x. 22519976

[49] Salis HM. 2011. The ribosome binding site calculator. Methods Enzymol 498:19–42. doi:10.1016/b978-0-12-385120-8.00002-4. 21601672

[50] Daddaoua A, Krell T, Ramos JL. 2009. Regulation of glucose metabolism in *Pseudomonas*: the phosphorylative branch and Entner-Doudoroff enzymes are regulated by a repressor containing a sugar isomerase domain. J Biol Chem 284:21360–21368. doi:10.1074/jbc.M109.014555. 19506074 PMC2755860

[51] Campilongo R, Fung RKY, Little RH, Grenga L, Trampari E, Pepe S, Chandra G, Stevenson CEM, Roncarati D, Malone JG. 2017. One ligand, two regulators and three binding sites: how KDPG controls primary carbon metabolism in *Pseudomonas*. PLoS Genet 13:e1006839. doi:10.1371/journal.pgen.1006839. 28658302 PMC5489143

[52] Silva-Rocha R, Martínez-García E, Calles B, Chavarría M, Arce-Rodríguez A, de las Heras A, Páez-Espino AD, Durante-Rodríguez G, Kim J, Nikel PI, Platero R, de Lorenzo V. 2013. The Standard European Vector Architecture (SEVA): a coherent platform for the analysis and deployment of complex prokaryotic phenotypes. Nucleic Acids Res 41:D666–D675. doi:10.1093/nar/gks1119. 23180763 PMC3531073

[53] Kim J, Oliveros JC, Nikel PI, de Lorenzo V, Silva-Rocha R. 2013. Transcriptomic fingerprinting of *Pseudomonas putida* under alternative physiological regimes. Environ Microbiol Rep 5:883–891. doi:10.1111/1758-2229.12090. 24249296

[54] Sadhu MJ, Moresco JJ, Zimmer AD, Yates JR, Rine J. 2014. Multiple inputs control sulfur-containing amino acid synthesis in *Saccharomyces cerevisiae*. Mol Biol Cell 25:1653–1665. doi:10.1091/mbc.E13-12-0755. 24648496 PMC4019496

[55] Igoillo-Esteve M, Cazzulo JJ. 2006. The glucose-6-phosphate dehydrogenase from *Trypanosoma cruzi*: its role in the defense of the parasite against oxidative stress. Mol Biochem Parasitol 149:170–181. doi:10.1016/j.molbiopara.2006.05.009. 16828178

[56] Mercaldi GF, Dawson A, Hunter WN, Cordeiro AT. 2016. The structure of a *Trypanosoma cruzi* glucose-6-phosphate dehydrogenase reveals differences from the mammalian enzyme. FEBS Lett 590:2776–2786. doi:10.1002/1873-3468.12276. 27391210

[57] Fuentealba M, Muñoz R, Maturana P, Krapp A, Cabrera R. 2016. Determinants of cofactor specificity for the glucose-6-phosphate dehydrogenase from *Escherichia coli*: simulation, kinetics and evolutionary studies. PLoS One 11:e0152403. doi:10.1371/journal.pone.0152403. 27010804 PMC4807051

[58] Rowland P, Basak AK, Gover S, Levy HR, Adams MJ. 1994. The three-dimensional structure of glucose 6-phosphate dehydrogenase from *Leuconostoc mesenteroides* refined at 2.0 Å resolution. Structure 2:1073–1087. doi:10.1016/S0969-2126(94)00110-3. 7881907

[59] Levy HR, Vought VE, Yin X, Adams MJ. 1996. Identification of an arginine residue in the dual coenzyme-specific glucose-6-phosphate dehydrogenase from *Leuconostoc mesenteroides* that plays a key role in binding NADP^+^ but not NAD^+^. Arch Biochem Biophys 326:145–151. doi:10.1006/abbi.1996.0058. 8579362

[60] Cacciapuoti AF, Lessie TG. 1977. Characterization of the fatty acid-sensitive glucose 6-phosphate dehydrogenase from *Pseudomonas cepacia*. J Bacteriol 132:555–563. doi:10.1128/JB.132.2.555-563.1977. 72065 PMC221896

[61] Kriventseva EV, Kuznetsov D, Tegenfeldt F, Manni M, Dias R, Simão FA, Zdobnov EM. 2019. OrthoDB v10: sampling the diversity of animal, plant, fungal, protist, bacterial and viral genomes for evolutionary and functional annotations of orthologs. Nucleic Acids Res 47:D807–D811. doi:10.1093/nar/gky1053. 30395283 PMC6323947

[62] Battistuzzi G, D'Urso M, Toniolo D, Persico GM, Luzzatto L. 1985. Tissue-specific levels of human glucose-6-phosphate dehydrogenase correlate with methylation of specific sites at the 3' end of the gene. Proc Natl Acad Sci U S A 82:1465–1469. doi:10.1073/pnas.82.5.1465. 3856275 PMC397283

[63] Pickl A, Schönheit P. 2015. The oxidative pentose phosphate pathway in the haloarchaeon *Haloferax volcanii* involves a novel type of glucose-6-phosphate dehydrogenase—the archaeal Zwischenferment. FEBS Lett 589:1105–1111. doi:10.1016/j.febslet.2015.03.026. 25836736

[64] Naeimpoor F, Mavituna F. 2000. Metabolic flux analysis in *Streptomyces coelicolor* under various nutrient limitations. Metab Eng 2:140–148. doi:10.1006/mben.2000.0146. 10935729

[65] Kiefer P, Heinzle E, Zelder O, Wittmann C. 2004. Comparative metabolic flux analysis of lysine-producing *Corynebacterium glutamicum* cultured on glucose or fructose. Appl Environ Microbiol 70:229–239. doi:10.1128/aem.70.1.229-239.2004. 14711646 PMC321251

[66] Daniels W, Bouvin J, Busche T, Rückert C, Simoens K, Karamanou S, van Mellaert L, Friðjónsson ÓH, Nicolai B, Economou A, Kalinowski J, Anné J, Bernaerts K. 2018. Transcriptomic and fluxomic changes in *Streptomyces lividans* producing heterologous protein. Microb Cell Fact 17:198. doi:10.1186/s12934-018-1040-6. 30577858 PMC6302529

[67] Gunnarsson N, Mortensen UH, Sosio M, Nielsen J. 2004. Identification of the Entner-Doudoroff pathway in an antibiotic-producing actinomycete species. Mol Microbiol 52:895–902. doi:10.1111/j.1365-2958.2004.04028.x. 15101992

[68] Tawfik DS. 2014. Accuracy-rate tradeoffs: how do enzymes meet demands of selectivity and catalytic efficiency? Curr Opin Chem Biol 21:73–80. doi:10.1016/j.cbpa.2014.05.008. 24954689

[69] Taymaz-Nikerel H, van Gulik WM, Heijnen JJ. 2011. *Escherichia coli* responds with a rapid and large change in growth rate upon a shift from glucose-limited to glucose-excess conditions. Metab Eng 13:307–318. doi:10.1016/j.ymben.2011.03.003. 21439400

[70] Tunio SA, Oldfield NJ, Ala'Aldeen DAA, Wooldridge KG, Turner DPJ. 2010. The role of glyceraldehyde 3-phosphate dehydrogenase (GapA-1) in *Neisseria meningitidis* adherence to human cells. BMC Microbiol 10:280. doi:10.1186/1471-2180-10-280. 21062461 PMC2994834

[71] Wistow GJ, Mulders JW, de Jong WW. 1987. The enzyme lactate dehydrogenase as a structural protein in avian and crocodilian lenses. Nature 326:622–624. doi:10.1038/326622a0. 3561501

[72] Felux AK, Spiteller D, Klebensberger J, Schleheck D. 2015. Entner-Doudoroff pathway for sulfoquinovose degradation in *Pseudomonas putida* SQ1. Proc Natl Acad Sci U S A 112:E4298–E4305. doi:10.1073/pnas.1507049112. 26195800 PMC4534283

[73] Akkaya Ö, Pérez-Pantoja D, Calles B, Nikel PI, de Lorenzo V. 2018. The metabolic redox regime of *Pseudomonas putida* tunes its evolvability toward novel xenobiotic substrates. mBio 9:e01512-18. doi:10.1128/mBio.01512-18. 30154264 PMC6113623

[74] Pérez-Pantoja D, Nikel PI, Chavarría M, de Lorenzo V. 2013. Endogenous stress caused by faulty oxidation reactions fosters evolution of 2,4-dinitrotoluene-degrading bacteria. PLoS Genet 9:e1003764. doi:10.1371/journal.pgen.1003764. 24009532 PMC3757077

[75] Khersonsky O, Tawfik DS. 2010. Enzyme promiscuity: a mechanistic and evolutionary perspective. Annu Rev Biochem 79:471–505. doi:10.1146/annurev-biochem-030409-143718. 20235827

[76] Guzmán GI, Utrilla J, Nurk S, Brunk E, Monk JM, Ebrahim A, Palsson BØ, Feist AM. 2015. Model-driven discovery of underground metabolic functions in *Escherichia coli*. Proc Natl Acad Sci U S A 112:929–934. doi:10.1073/pnas.1414218112. 25564669 PMC4311852

[77] Weimer A, Kohlstedt M, Volke DC, Nikel PI, Wittmann C. 2020. Industrial biotechnology of *Pseudomonas putida*: advances and prospects. Appl Microbiol Biotechnol 104:7745–7766. doi:10.1007/s00253-020-10811-9. 32789744 PMC7447670

[78] Nikel PI, Pérez-Pantoja D, de Lorenzo V. 2016. Pyridine nucleotide transhydrogenases enable redox balance of *Pseudomonas putida* during biodegradation of aromatic compounds. Environ Microbiol 18:3565–3582. doi:10.1111/1462-2920.13434. 27348295

[79] Grant CM. 2008. Metabolic reconfiguration is a regulated response to oxidative stress. J Biol 7:1. doi:10.1186/jbiol63. 18226191 PMC2246036

[80] Ralser M, Wamelink MM, Kowald A, Gerisch B, Heeren G, Struys EA, Klipp E, Jakobs C, Breitenbach M, Lehrach H, Krobitsch S. 2007. Dynamic rerouting of the carbohydrate flux is key to counteracting oxidative stress. J Biol 6:10. doi:10.1186/jbiol61. 18154684 PMC2373902

[81] Lessie TG, Phibbs PV. 1984. Alternative pathways of carbohydrate utilization in pseudomonads. Annu Rev Microbiol 38:359–388. doi:10.1146/annurev.mi.38.100184.002043. 6388497

[82] Calero P, Nikel PI. 2019. Chasing bacterial *chassis* for metabolic engineering: a perspective review from classical to non-traditional microorganisms. Microb Biotechnol 12:98–124. doi:10.1111/1751-7915.13292. 29926529 PMC6302729

[83] Sánchez-Pascuala A, de Lorenzo V, Nikel PI. 2017. Refactoring the Embden-Meyerhof-Parnas pathway as a whole of portable GlucoBricks for implantation of glycolytic modules in Gram-negative bacteria. ACS Synth Biol 6:793–805. doi:10.1021/acssynbio.6b00230. 28121421 PMC5440799

[84] Sánchez-Pascuala A, Fernández-Cabezón L, de Lorenzo V, Nikel PI. 2019. Functional implementation of a linear glycolysis for sugar catabolism in *Pseudomonas putida*. Metab Eng 54:200–211. doi:10.1016/j.ymben.2019.04.005. 31009747

[85] Durante-Rodríguez G, de Lorenzo V, Nikel PI. 2018. A post-translational metabolic switch enables complete decoupling of bacterial growth from biopolymer production in engineered *Escherichia coli*. ACS Synth Biol 7:2686–2697. doi:10.1021/acssynbio.8b00345. 30346720

[86] Sambrook J, Russell DW. 2001. Molecular cloning: a laboratory manual, 3rd ed, Cold Spring Harbor Laboratory Press, Cold Spring Harbor, NY.

[87] Choi KH, Kumar A, Schweizer HP. 2006. A 10-min method for preparation of highly electrocompetent *Pseudomonas aeruginosa* cells: application for DNA fragment transfer between chromosomes and plasmid transformation. J Microbiol Methods 64:391–397. doi:10.1016/j.mimet.2005.06.001. 15987659

[88] Bradford MM. 1976. A rapid and sensitive method for the quantitation of microgram quantities of protein utilizing the principle of protein-dye binding. Anal Biochem 72:248–254. doi:10.1016/0003-2697(76)90527-3. 942051

[89] Segel IH. 2014. Enzyme kinetics: behavior and analysis of rapid equilibrium and steady-state enzyme systems. Wiley, Oxford, United Kingdom.

[90] Cornish-Bowden A. 1975. The use of the direct linear plot for determining initial velocities. Biochem J 149:305–312. doi:10.1042/bj1490305. 1180900 PMC1165624

[91] Kuzmic P. 1996. Program DYNAFIT for the analysis of enzyme kinetic data: application to HIV proteinase. Anal Biochem 237:260–273. doi:10.1006/abio.1996.0238. 8660575

[92] Edgar RC. 2004. MUSCLE: multiple sequence alignment with high accuracy and high throughput. Nucleic Acids Res 32:1792–1797. doi:10.1093/nar/gkh340. 15034147 PMC390337

[93] Jun SR, Sims GE, Wu GA, Kim SH. 2010. Whole-proteome phylogeny of prokaryotes by feature frequency profiles: an alignment-free method with optimal feature resolution. Proc Natl Acad Sci U S A 107:133–138. doi:10.1073/pnas.0913033107. 20018669 PMC2806744

[94] Crooks GE, Hon G, Chandonia JM, Brenner SE. 2004. WebLogo: a sequence logo generator. Genome Res 14:1188–1190. doi:10.1101/gr.849004. 15173120 PMC419797

[95] Belda E, van Heck RGA, López-Sánchez MJ, Cruveiller S, Barbe V, Fraser C, Klenk HP, Petersen J, Morgat A, Nikel PI, Vallenet D, Rouy Z, Sekowska A, Martins dos Santos VAP, de Lorenzo V, Danchin A, Médigue C. 2016. The revisited genome of *Pseudomonas putida* KT2440 enlightens its value as a robust metabolic *chassis*. Environ Microbiol 18:3403–3424. doi:10.1111/1462-2920.13230. 26913973

