# Supplementary figures and images for: Cofactor Specificity of Glucose-6-Phosphate Dehydrogenase Isozymes in Pseudomonas putida Reveals a General Principle Underlying Glycolytic Strategies in Bacteria
Source: mSystems. 2021 Mar 16;6(2):e00014-21. doi: 10.1128/mSystems.00014-21 (PMC8546961; doi:10.1128/mSystems.00014-21)

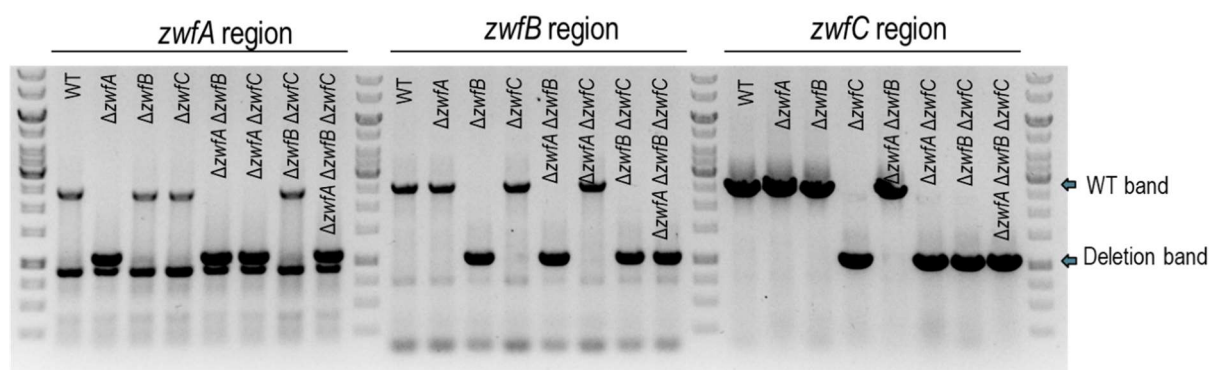

Supplement: FIG S1 [file msystems.00014-21-sf001.pdf]

Citrate

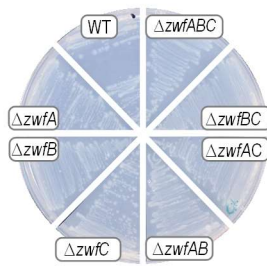

Glucose

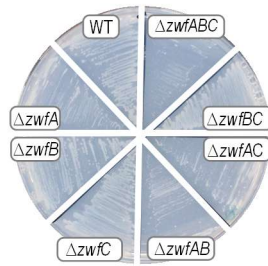

Fructose

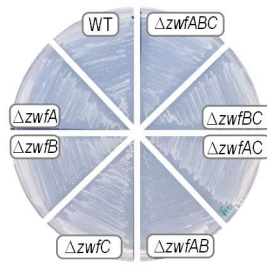

Ribose (180 h)

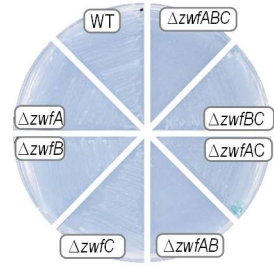

Supplement: FIG S2 [file msystems.00014-21-sf002.pdf]

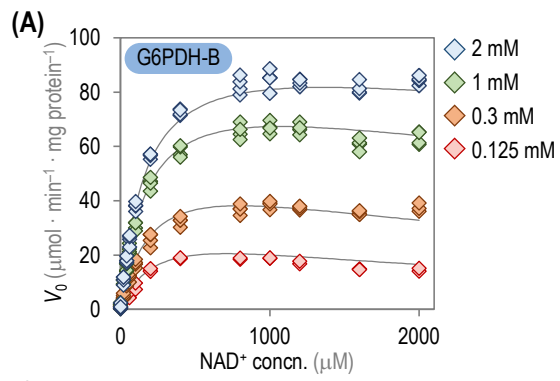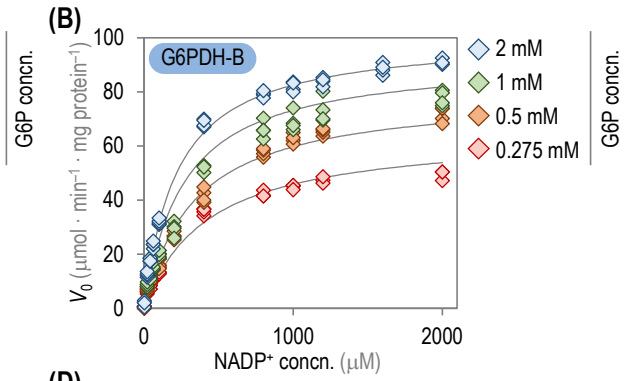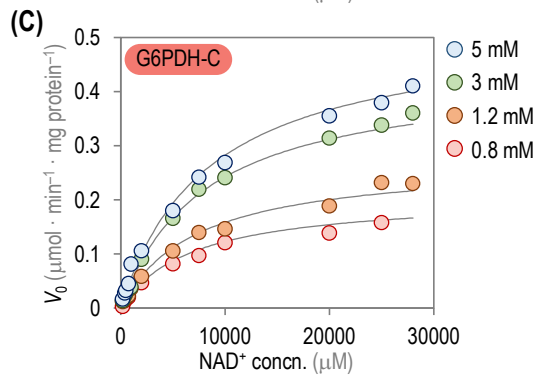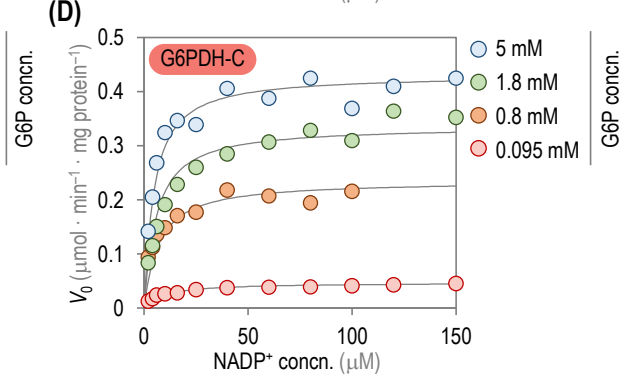

Supplement: FIG S3 [file msystems.00014-21-sf003.pdf]

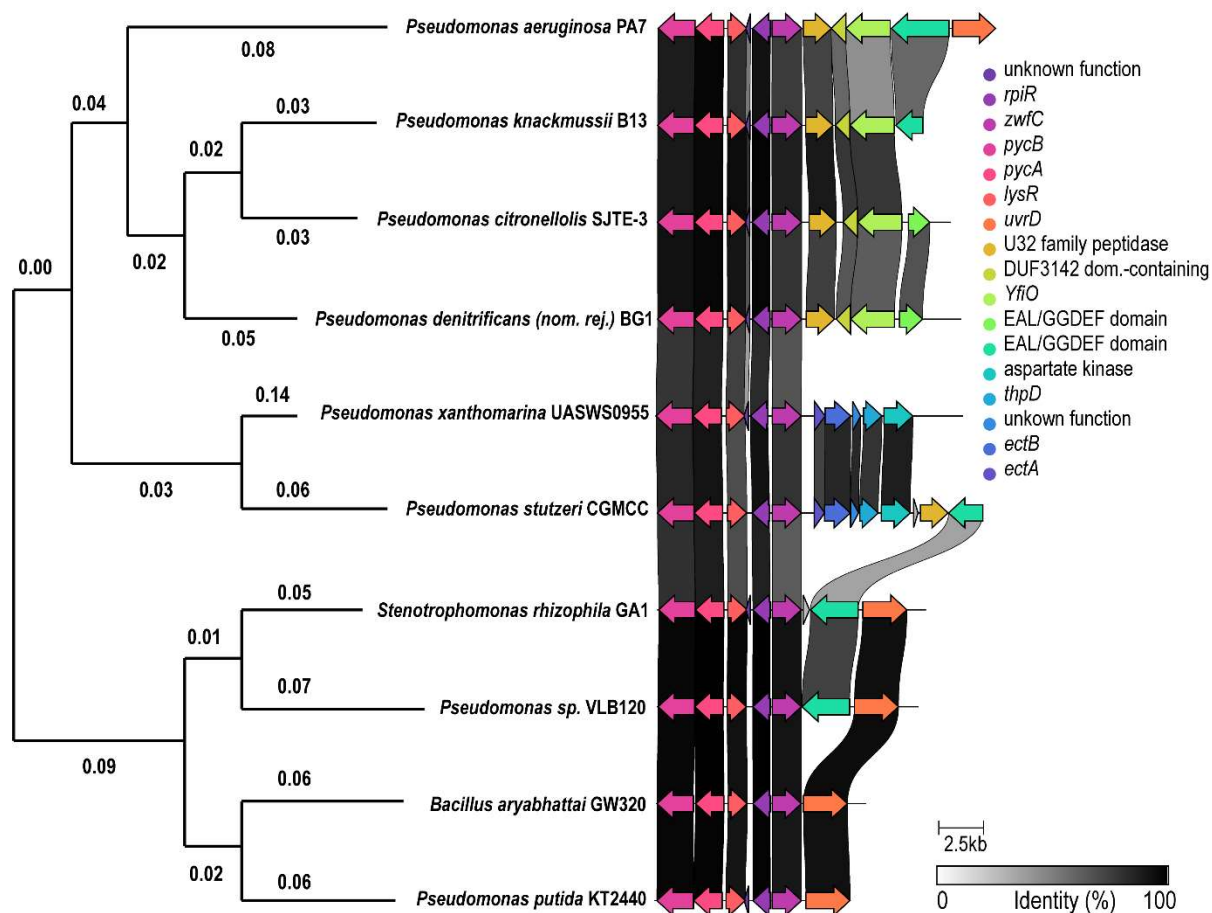

Supplement: FIG S4 [file msystems.00014-21-sf004.pdf]

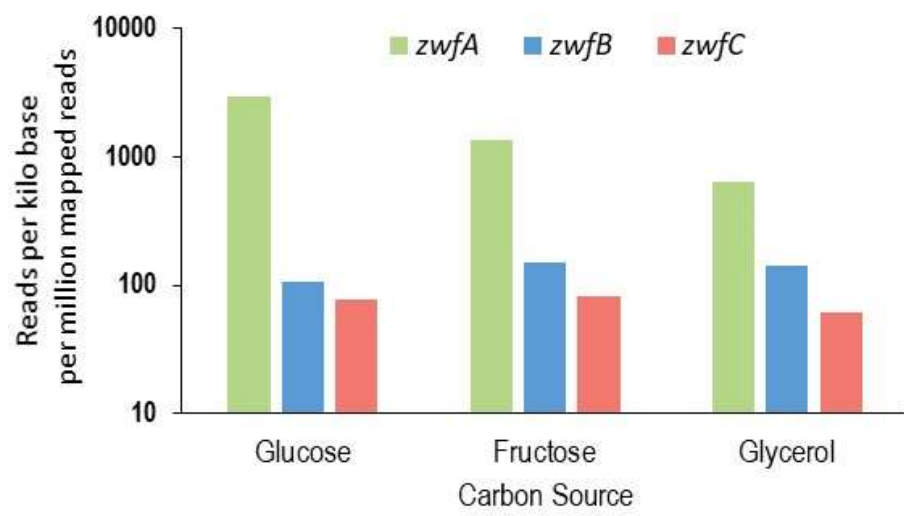

Supplement: FIG S5 [file msystems.00014-21-sf005.pdf]

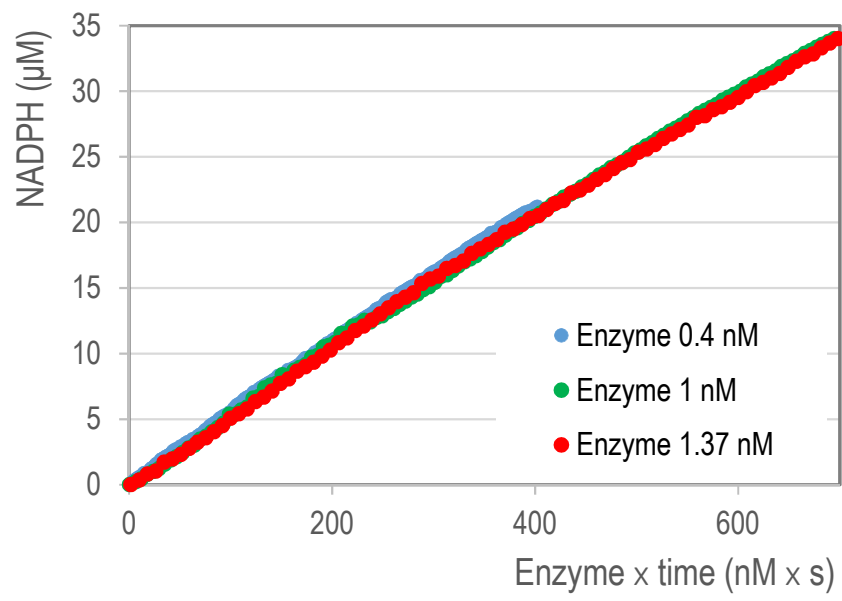

Supplement: FIG S7 [file msystems.00014-21-sf007.pdf]
