# Supplementary material for: Cofactor Specificity of Glucose-6-Phosphate Dehydrogenase Isozymes in Pseudomonas putida Reveals a General Principle Underlying Glycolytic Strategies in Bacteria
Source: mSystems. 2021 Mar 16;6(2):e00014-21. doi: 10.1128/mSystems.00014-21 (PMC8546961; doi:10.1128/mSystems.00014-21)
Supplement: TABLE S1 [file msystems.00014-21-st001.pdf]

Table S1. Bacterial strains and plasmids used in this study.[a]

Name	Relevant characteristics[a]	Source or reference
**Bacterial strain**		
E. coli DH5α	Cloning host; F^- λ^- *endA1 glnX44*(AS) *thiE1 recA1 relA1 spoT1 gyrA96*(Nal^R) *rfbC1 deoR nupG* Φ80(*lacZ*Δ*M15*) Δ(*argF-lac*)*U169 hsdR17*(r_K^- m_K^+)	(1)
E. coli DH5α λ*pir*	Cloning host; λ*pir* lysogen derivative of strain DH5α	(2)
E. coli BL21DE3	Expression host, *fhuA2 [lon] ompT gal* (λ_{DE3}) *[dcm]* Δ*hsdS* [λ_{DE3} = λ *sBamHIo* Δ*EcoRI-B int::(lacI::*P_{lacUV5}*::T7 gene1) i21* Δ*nin5]*	New England Biolabs
P. putida KT2440	Wild-type strain; derivative of *P. putida* mt-2 (3) cured of the TOL plasmid pWW0	(4)
**Plasmid**		
pEMG	Suicide vector used for deletions in Gram-negative bacteria; *oriT*, *traJ*, *lacZ*α, *ori*(R6K); Km^R	(5)
pSEVA212s	Suicide vector used for deletions in Gram-negative bacteria; *oriT*, *traJ*, *lacZ*α, *ori*(R6K); Km^R	(6)
pSEVA628s	Helper plasmid; *oriV*(RK2), XylS/*Pm*→*I-SceI*; Gm^R	(7)
pS212s·Δ*zwfA*	Derivative of vector pEMG, carrying HRs to delete *zwfA* (*PP_1022*)	This work
pEMG·Δ*zwfB*	Derivative of vector pEMG, carrying HRs to delete *zwfB* (*PP_4042*)	This work
pEMG·Δ*zwf*	Derivative of vector pEMG, carrying HRs to delete *zwf* (*PP_5351*)	This work
pEMG·Δ*hexR*	Derivative of vector pEMG, carrying HRs to delete *hexR* (*PP_1021*)	This work
pEMG·Δ*rpiR*	Derivative of vector pEMG, carrying HRs to delete *rpiR* (*PP_5350)*	This work
pSEVA227Y	Cloning vector; *oriV*(RK2), promoter-less *yfp*; Km^R	(8)
pS22T·P_{zwfA}	Derivative of vector pS227Y with 302-bp-long genomic region upstream of *zwfA*	This work
pS22T·P_{zwfC}	Derivative of vector pS227Y with 278-bp-long genomic region upstream of *zwf*	This work
pET28a	Expression vector, *oriV*(pBR322), P_{T7}, Km^R	Novagen
pET28a::Zwf	Derivative of vector pET28a, P_{T7}→*zwf*	This work
pET28a::ZwfB	Derivative of vector pET28a, P_{T7}→*zwfB*	This work

[a] *Antibiotic markers*: Gm, gentamicin; Km, kanamycin; Nal, nalidixic acid; and Str, streptomycin.

REFERENCES

1. **Meselson M, Yuan R.** 1968. DNA restriction enzyme from *E. coli*. Nature **217:**1110-1114. http://dx.doi.org/10.1038/2171110a0

2. **Platt R, Drescher C, Park SK, Phillips GJ.** 2000. Genetic system for reversible integration of DNA constructs and *lacZ* gene fusions into the *Escherichia coli* chromosome. Plasmid **43:**12-23. http://dx.doi.org/https://doi.org/10.1006/plas.1999.1433

3. **Worsey MJ, Williams PA.** 1975. Metabolism of toluene and xylenes by *Pseudomonas putida* (*arvilla*) mt-2: evidence for a new function of the TOL plasmid. J. Bacteriol. **124:**7-13.

4. **Bagdasarian M, Lurz R, Rückert B, Franklin FCH, Bagdasarian MM, Frey J, Timmis KN.** 1981. Specific purpose plasmid cloning vectors. II. Broad host range, high copy number, RSF1010-derived vectors, and a host-vector system for gene cloning in *Pseudomonas*. Gene **16:**237-247. http://dx.doi.org/10.1016/0378-1119(81)90080-9

5. **Martínez-García E, de Lorenzo V.** 2011. Engineering multiple genomic deletions in Gram-negative bacteria: analysis of the multi-resistant antibiotic profile of *Pseudomonas putida* KT2440. Environ. Microbiol. **13:**2702-2716. http://dx.doi.org/10.1111/j.1462-2920.2011.02538.x

6. **Silva-Rocha R, Martínez-García E, Calles B, Chavarría M, Arce-Rodríguez A, de las Heras A, Páez-Espino AD, Durante-Rodríguez G, Kim J, Nikel PI, Platero R, de Lorenzo V.** 2013. The Standard European Vector Architecture (SEVA): a coherent platform for the analysis and deployment of complex prokaryotic phenotypes. Nucleic Acids Res. **41:**D666-D675. http://dx.doi.org/10.1093/nar/gks1119

7. **Aparicio T, Jensen SI, Nielsen AT, de Lorenzo V, Martínez-García E.** 2016. The Ssr protein (*T1E_1405*) from *Pseudomonas putida* DOT-T1E enables oligonucleotide-based recombineering in platform strain *P. putida* EM42. Biotechnol. J. **11:**1309-1319. http://dx.doi.org/10.1002/biot.201600317

8. **Martínez-García E, Aparicio T, Goñi-Moreno A, Fraile S, de Lorenzo V.** 2015. SEVA 2.0: an update of the Standard European Vector Architecture for de-/re-construction of bacterial functionalities. Nucleic Acids Res. **43:**D1183-D1189. http://dx.doi.org/10.1093/nar/gku1114
