# Supplementary material for: Cofactor Specificity of Glucose-6-Phosphate Dehydrogenase Isozymes in Pseudomonas putida Reveals a General Principle Underlying Glycolytic Strategies in Bacteria
Source: mSystems. 2021 Mar 16;6(2):e00014-21. doi: 10.1128/mSystems.00014-21 (PMC8546961; doi:10.1128/mSystems.00014-21)
Supplement: FIG S6 [file msystems.00014-21-sf006.pdf]

```
zwfB        MTKQTLAAAPPCTLFLFGANGDLVKRLLMPALYNLSRDGLLDRNLRIVGVDHNPASAEDF  60
zwfA        ---MAAISVEPCTFALFGALGDLALRKLFPALYQLDRANLLHPDTRLLALAREVGSAQEH  57
zwfC        --VESGGLTIPCDILVFGGTGDLALHKLLPALYHLFREARLNPAVRVIALARRNLPRIDY  58
                :   . ** : :**. ***. : *:****:* *   *.   *::.: :.     :.

zwfB        AARLHAFMVERDKGGEGSAKCLDEKLWARLAKRLDYQTGDFLDPATYQALARRIDKTRHG  120
zwfA        LDSI-----EAHLRRHVPEADIEPAALGRFLARLNYQHLDFLQPEGYQALAEQ--LPGEL  110
zwfC        LKLA-----ERHCRAQIARSDFDEEVWQRFSARVDYFPMDASQSADFGRLARYLGEPGGL  113
                     * .   .     ::     *:  *::*   *  :   :  **.

zwfB        NAIFYLATSPRFFPEVAQRLGQAGLLDESAGGFRRVVVEKPFGTDLASAEALNACLLKVM  180
zwfA        PLIAYFATAAAVYGAICENLDKVGLAA-----RTRVVLEKPIGHDLESSRRVNDAVARFF  165
zwfC        TRIFYLATAPNLFVPIANHLRIAGLADT----EARIVLEKPIGHSLESATAINEAIGTVF  169
              * *:**:  .:  :.:.*  .**         *:*:***:* .* *:  :* .:  .:

zwfB        GERQIYRIDHYLGKETVQNILVSRFSNGLFESFWNNHYIDHVQITAAETVGVETRGAFYD  240
zwfA        PESRVYRIDHYLGKETVQNLIALRFANSLFETQWNQNSISHVEITVAEKVGIEGRWGYFD  225
zwfC        EERQVFRIDHYLGKETVQNLMALRFANALLEPVWRNNQVDHVQISVCETLGVENRGAYYD  229
             * :::*************::. **:*.*:*  *.:: :.**:*:..*.:*:* * .::*

zwfB        STGALRDMVPNHLFQLLAMVAMEPPAAFGADAVRGEKAKVVGAIRPWSAKMAQKNSVRGQ  300
zwfA        KAGQLRDMIQNHLLQLLCLIAMDPPSELSADAIRDEKVKVLKALAPITGDGLSTSVVRGQ  285
zwfC        RAGAIRDMLQNHLLQLLCLVAMEPPAQFEAEAVRDEKVKILRALKPITGQDVQDKTVRGQ  289
             :* :***: ***:***.::**:**: : *:*:*.**.*:: *: * :..  . . ****

zwfB        YRAGKQGRKPLPGYRQEPNVAPDSQTETYVALKVMIDNWRWAGVPFYLRTGKRMSVRDTE  360
zwfA        YIAGYSEGKPVPGYLEEDNANAQSDTETFVALRADIRNWRWSGVPFYLRTGKRMPQKLSQ  345
zwfC        YGAGRIGGQEVPAYYFEKDVDNDSDTETFVAIEAHIDNWRWAGVPFYLRTGKRMARRASQ  349
            * **    : :*.*  * :.  :*:***:**:.. * ****:************  : ::

zwfB        IAICFKPAPYAQFRESELERPKPNYLKIQIQPNEGMWFDLQAKRPGPE--LVMENVELGF  418
zwfA        IVIHFKETPHYIFAPEQRL-QIGNKLIIRLQPDEGISLRVMTKEQGLDKGMQLRSGPLQL  404
zwfC        IVIQFKPVPHELFSGGQV-----NQLLIQLQPDERISLRMMTKSPG--KGMRLEPVDLDL  402
            *.* ** .*:  *   :      * * *::**:* : : : :*  *    : :.   * :

zwfB        AYKDFFKM-TPATGYETLIYDCLTGDQTLFQRADNIENGWRAVQPFLDAWAQGGE-VHEY  476
zwfA        NFSDAWRSARIPDAYERLLLEVMRGNQNLFVRKDEIEYAWKWCDQLIAGWRNAGDAPKPY  464
zwfC        NLAQVFSQTRRWEAYERLLLDVLEGDSTLFMRRDEVEAAWAWVDPIIKGCEEHFQAPRPY  462
               : :       .** *: : : *:..** * *::* .*   : :: .  :  : :  *

zwfB        SAGEDGPEAGNELLTRDKREWHRLG*    501
zwfA        AAGSWGPMSSIALITRDGRAWYGDI*    489
zwfC        PAGSFGPEQANSLLARHGRHWHG*--    485
             **. **  .  *::*. * *:
```
