# Supplementary material for: Cofactor Specificity of Glucose-6-Phosphate Dehydrogenase Isozymes in Pseudomonas putida Reveals a General Principle Underlying Glycolytic Strategies in Bacteria
Source: mSystems. 2021 Mar 16;6(2):e00014-21. doi: 10.1128/mSystems.00014-21 (PMC8546961; doi:10.1128/mSystems.00014-21)
Supplement: TABLE S2 [file msystems.00014-21-st002.pdf]

Table S2. Oligonucleotides used in this study.[a]

Oligonucleotide	Sequence (5'→3')	Use
P_{ZwfA}_EcoRI-F	GCG C*GA ATT C*GC GGG TTG AGC AGG ATG ACT T	Amplification of promoter region upstream of *zwfA* and *zwf*
P_{ZwfA}_BamHI-R	TAG A*GG ATC C*GA TGT TGG TTC TTT CCT AAA GTT GGT CT	
P_{Zwf}_EcoRI-F	GCG C*GA ATT C*GG AAG CCC GAG CAG CCA AT	
P_{Zwf}_BamHI-R	AGA *GGA TCC* GTT CGT TTA ACT GGG CGG GTT G	
ZwfC_US-HindIII-F	AAA *AAG CTT* CGC CAA AGC CAT AGA ACT CTA CCC	Deletion of *zwf* (*PP_5351*)
ZwfC_US-R	GAT TAA AGA TCG CGC CAT CGC GCA GGC CAT GTT CGT TTA ACT GGG CGG GTT GG	
ZwfC_DS-F	ATG GAC GCC AAC CCG CCC AGT TAA ACG AAC ATG GCC TGC GCG ATG GC	
ZwfC_DS-BamHI-R	AAA *GGA TCC* GGC CGT GGA AGG TGC CTA CC	
ZwfA_US-BamHI-F	AAA *GGA TCC* GCA GGC AGC GGC GTG G	Deletion of *zwfA* (*PP_1022*)
ZwfA_US-R	CCT AGA CCA ACT TTA GGA AAG AAC CAA CAT CTG GGG GCC GAT GAG CTC GA	
ZwfA_DS-F	GAT CAG TGC AAT CGA GCT CAT CGG CCC CCA GAT GTT GGT TCT TTC CTA AAG TTG GTC T	
ZwfA_DS-HindIII-R	AAA *AAG CTT* GGG TGT AGG CGG CCG G	
ZwfB_US-BamHI-F	AAA *GGA TCC* CCG GTC AGG GCC GGC GA	Deletion of *zwfB* (*PP_4042*)
ZwfB_US-R	GGT GGC CAC GTC GAG AAG AAA GGC GAA TGA GAG GTT TGC AAG CCT GTG CCG	
ZwfB_DS-F	CGA AAG GGC CGG CAC AGG CTT GCA AAC CTC TCA TTC GCC TTT CTT CTC GAC GTG	
ZwfB_DS-SalI-R	AAA *GTC GAC* CGT ACC CAT GGC CGC AGC	
ZwfB_NdeI-F	CTT ATC *CAT ATG* ACT AAA CAG ACC CTT GCG	Amplification of *zwfB*
ZwfB_BamHI-R	CTG ATT *GGA TCC* TCA CCC CAA CCG GTG CCA CTC C	
Zwf_NdeI-F	CTT ATC *CAT ATG* GAG AGT GGT GGT TTG ACT ATT CC	Amplification of *zwf*
Zwf_BamHI-R	CAA TAT *GGA TCC* TCA GCC ATG CCA GTG CCT GCC ATG	
*hexR*_US-BamHI-F	AA*G GAT CC*G CTC GCC CGG CAG CTG CTC	Deletion of *hexR* (*PP_1021*)
*hexR*_US-R	CTC AGT TGA GGT CGT CCA CGC GGT CCA TGG GTG TGT C	

*hexR*_DS-F	CCC ATG GAC CGC GTG GAC GAC CTC AAC TGA GCC GGT GC	Deletion of *hexR* (*PP_1021*)
*hexR*_DS-EcoRI-R	AA*G AAT TC*G CGG CTG GAC CTG TGC GAC TGG	
*rpiR*_US-BamHI-F†	AGA TCC UGT CCG GCA ATG CGC AGG T	Deletion of *rpiR* (*PP_5350*)
*rpiR*_US-R†	AGG TTG AAU AGG CGG GAC TCG GTT ATG AT	
*rpiR*_DS-F†	AGG TCG ACU CCA AGG CCA AGC GGC AGT CA	
rpiR _DS-SalI-R†	ATT CAA CCU GTA CTG GCC TGT TCG CGG GC	

[a] Oligonucleotides designed for *USER* cloning are indicated with a † symbol.
